# Hemin-catalyzed oxidative oligomerization of *p*-aminodiphenylamine (PADPA) in the presence of aqueous sodium dodecylbenzenesulfonate (SDBS) micelles†

**DOI:** 10.1039/d2ra02198f

**Published:** 2022-05-03

**Authors:** Nemanja Cvjetan, Reinhard Kissner, Danica Bajuk-Bogdanović, Gordana Ćirić-Marjanović, Peter Walde

**Affiliations:** Department of Materials, Laboratory for Multifunctional Materials, ETH Zürich Vladimir-Prelog-Weg 5 8093 Zürich Switzerland peter.walde@mat.ethz.ch; Department of Chemistry and Applied Biosciences, Laboratory of Inorganic Chemistry Vladimir-Prelog-Weg 2 8093 Zürich Switzerland; Faculty of Physical Chemistry, University of Belgrade Studentski trg 12-16 11158 Belgrade Serbia

## Abstract

In a previous report on the enzymatic synthesis of the conductive emeraldine salt form of polyaniline (PANI-ES) in aqueous solution using PADPA (*p*-aminodiphenylamine) as monomer, horseradish peroxidase isoenzyme C (HRPC) was applied as a catalyst at pH = 4.3 with H_2_O_2_ as a terminal oxidant. In that work, anionic vesicles were added to the reaction mixture for (i) guiding the reaction to obtain poly(PADPA) products that resemble PANI-ES, and for (ii) preventing product precipitation (known as the “template effect”). In the work now presented, instead of native HRPC, only its prosthetic group ferric heme *b* (= hemin) was utilized as a catalyst, and micelles formed from SDBS (sodium dodecylbenzenesulfonate) served as templates. For the elaborated optimal reaction conditions, complementary UV/vis/NIR, EPR, and Raman spectroscopy measurements clearly showed that the reaction mixture obtained after completion of the reaction contained PANI-ES-like products as dominating species, very similar to the products formed with HRPC as catalyst. HEPES (4-(2-hydroxyethyl)-1-piperazineethanesulfonate) was found to have a positive effect on the reaction rate as compared to dihydrogenphosphate. This work is the first on the template-assisted formation of PANI-ES type products under mild, environmentally friendly conditions using hemin as a cost-effective catalyst.

## Abstract 2

Polyaniline emeraldine salt-type products were synthesized under mild, environmentally friendly conditions using hemin as a cost-effective catalyst, *p*-aminodiphenylamine (PADPA) as a monomer, and micelles formed from SDBS as templates.

## Graphical abstract

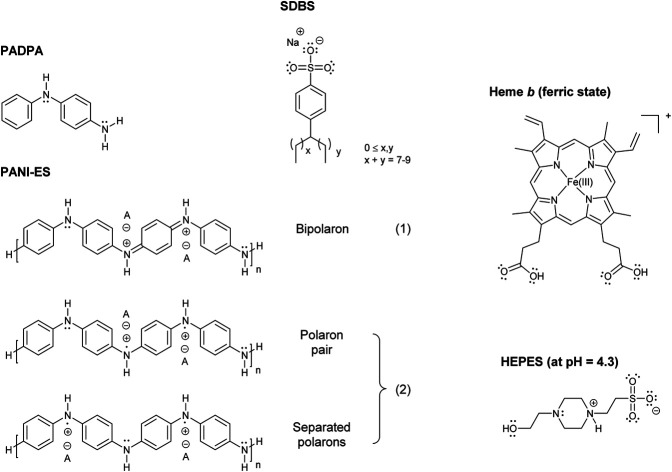

## 1. Introduction

**Fig. 1:**
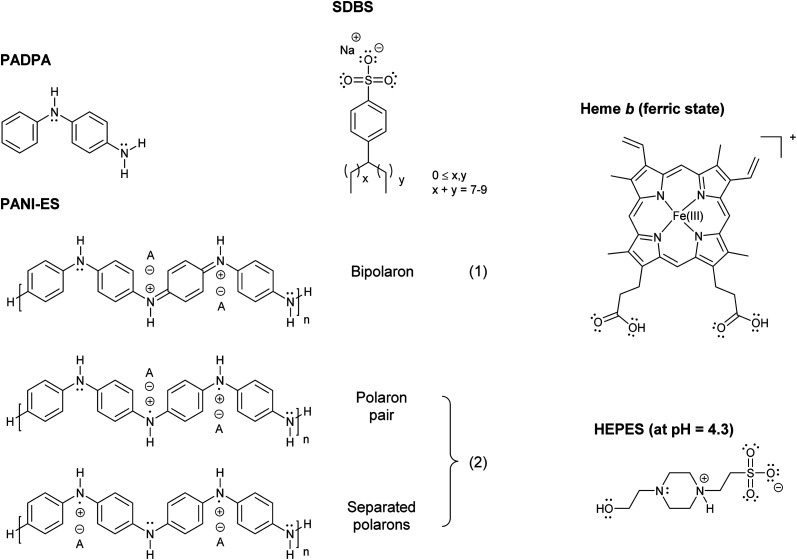
Chemical structures of PADPA (= *p*-aminodiphenylamine); PANI-ES, the emeraldine salt form of linear polyaniline in the bipolaron (1) and conductive polaron (2) states; SDBS (= sodium dodecylbenzenesulfonate), hard type;^15^ heme *b* in its ferric state (= Fe(iii)protoporphyrin IX = por Fe^III^ = hemin) with undissociated carboxylic acid groups at pH = 4.3;^27^ and HEPES (= 4-(2-hydroxyethyl)-1-piperazineethansulfonic acid) in the zwitterionic form, which dominates at pH = 4.3 (deprotonated sulfonic acid and protonated N-atom close to the sulfonate group).^55^

In a series of previous studies, it was shown that HRPC (horseradish peroxidase isoenzyme C) can be successfully used in the presence of hydrogen peroxide (H_2_O_2_) as a terminal oxidant in aqueous solution at pH = 4.3 and at room temperature for the synthesis of oligomeric and polymeric products rich in the conductive emeraldine salt form of linear polyaniline (PANI-ES) (Fig. 1). As monomers, either aniline,^1–4^ the aniline dimer *p*-aminodiphenylamine (PADPA)^5^ (Fig. 1), or mixtures of aniline and PADPA^6^ were used. Moreover, for obtaining reaction products that contain substantial amounts of the ideal PANI-ES repeating unit, *i.e.*, half oxidized and protonated linear tetraaniline (Fig. 1), the presence of aggregates of anionic amphiphiles (mainly micelles or vesicles)^1,7^ or anionic polyelectrolytes^8^ as “templates” was shown to be essential. One of the roles these templates play in the reaction mixture is to accumulate the monomers by electrostatic and hydrophobic interactions, thereby increasing their local concentration. As a consequence, once the monomers are oxidized by HRPC/H_2_O_2_, the follow-up reactions are influenced by the templates in a positive way, leading to a desired coupling of the oxidized monomers so that finally PANI-ES repeating units are obtained.^4,6,9–12^ The use of anionic vesicles from AOT (the sodium salt of bis(2-ethylhexyl) sulfosuccinate) turned out to be particularly suitable for these HRPC-catalyzed reactions,^1,5,13^ but the reactions also proceeded well with micelles formed from SDBS (sodium dodecylbenzenesulfonate) (Fig. 1) using aniline as monomer.^14^ In the presence of SDBS micelles, however, HRPC is less stable than in the presence of AOT vesicles.^14,15^

The idea behind the work presented now was to try to synthesize PANI-ES products from PADPA as monomer in the presence of SDBS micelles by using instead of HRPC its prosthetic group, ferric heme *b*‡‡Although it has been suggested to use capital letters for specifying isolated, free hemes, *e.g.* heme *B*, and lowercase letters for hemes bound or associated to proteins (*i.e.*, heme *b*), in this work we do not make this distinction and always use “heme *b*” for both cases.^78^ The extent of amphiphilicity of heme *b* depends on the degree of dissociation of the two carboxylic acids. At the pH value of the aqueous solutions used in this work, it is likely that the two carboxylic acids are not ionized at all. (Fig. 1).

In ferric heme *b*, Fe^3+^ is coordinated to the four nitrogen atoms of protoporphyrin IX (Fig. 1). Other designations and abbreviations for ferric heme *b* are iron(iii) protoporphyrin IX, Fe(iii)PPIX, or porFe^III^ (“por” standing for “protoporphyrin IX”). Ferric heme *b* is also known as hemin.^16^ The commercially available hemin form contains a chloride ion at the 5^th^ coordination position (porFe^III^-Cl).

The SDBS micelles used should play two essential roles in the reaction. They should not only serve as templates for the reaction, but also as a host for hemin by providing a local environment that ensures that hemin is in a catalytically active state. It is well known that heme *b* can form aggregates in an aqueous medium depending on the experimental conditions: π–π dimers, μ-oxo dimers, or large stacks of μ-oxo dimers.^17–19^ However, since only monomeric heme *b* is catalytically active, like at the active site of heme peroxidases^20,21^ and of other heme proteins,^22–24^ binding of hemin to the micelles must prevent hemin aggregation. Therefore, the challenges of the work were to investigate (i) whether suitable conditions can be found under which hemin in the presence of anionic SDBS micelles is in catalytically active (monomeric) state towards PADPA as substrate, and (ii) whether PANI-ES-like oligo- or poly(PADPA) products are formed under these conditions.

The use of micelles for hosting heme *b* was studied before, although investigations of the average localization of heme *b* in micelles are still rare.^25–27^ Based on previous reports, it is evident that only in the presence of certain types of micelle-forming surfactants monomeric hemin is obtained.^17^ So far, sodium dodecylsulfate (SDS) was most commonly used.^25–30^

Other possibilities for obtaining monomeric hemin in aqueous medium are to work at very low concentration,^18–20^ to use human serum albumin, to which heme *b* binds in monomeric state at one of the hydrophobic binding sites,^31–33^ or to form complexes with guanine (G)-rich DNAs (so-called G-quadruplex-DNAs).^34–38^ In the latter case, the activity of such complexes towards aniline was reported by Shen *et al.* (2014).^39^ Compared to G-quadruplex-DNAs or human serum albumin, however, SDBS micelles are not only simpler and cheaper hosts for hemin, but they can also serve as templates for the reaction (see above).

When hemin is placed in a non-protein environment, as in this work with SDBS micelles, a logical question arises: does the hemin/SDBS micelle system follow the same catalytic cycle as heme peroxidases do (but perhaps less efficiently)? Or, do alternative mechanisms play a role? Scheme S-1, ESI,† provides an overview of the peroxidase cycle of HRPC.^1,20,40–48^ In brief, the two-electron oxidation by H_2_O_2_ of porFe^III^ in the resting state of HRPC is followed by two one-electron oxidations of a reducing substrate, which is PADPA in the present case. Depending on the reducing substrate, non-enzymatic follow-up reactions lead to the final product(s), *i.e.*, oligo- or/and poly(PADPA).^1,5^

Regardless of the mechanism by which PADPA is oxidized, the performance of the hemin/SDBS micelle system for the oxidation of PADPA to PANI-ES-like products was evaluated using the following criteria: (i) presence of spectroscopic features of the reaction products that are characteristic for the formation of PANI-ES; (ii) presence of high amounts of favorable PANI-ES products; (iii) high “quality” of the formed products in terms of amount of linear PANI-ES products *vs.* amount of unfavorable products containing branches or phenazine-type units; and (iv) no precipitation of the products formed. Therefore, the aqueous reaction mixtures were analyzed by three complementary methods, UV/vis/NIR, EPR, and *in situ* Raman spectroscopy measurements. Characteristic absorption bands for PANI-ES-like products are in the near-infrared (NIR) and visible (vis) regions of the spectrum, at *λ*_max_ ≈ 800–1100 nm, assigned to the π → polaron transition,^49^ and at *λ*_max_ ≈ 420 nm, assigned to the polaron → π* transition,^49^ with low absorption at *λ* ≈ 500–600 nm, indicative for the absence of extensive branching and absence of phenazine-type units.^5,8^ Due to the presence of unpaired electrons, conductive PANI-ES products in their polaron form (Fig. 1) have an EPR spectrum.^50–52^ Raman bands originating from C–N˙^+^ stretching vibrations of the polaron form of PANI-ES are at wavenumbers (*ν̃*) in the range of 1320–1380 cm^−1^.^11,53^

As a part of the work with the hemin/SDBS micelle system, we also explored the possible effect the type of salt used for the preparation of the aqueous pH = 4.3 solution might have on the rate of reaction and on the product distribution.§§For the pH = 4.3 conditions used for the reaction, the “buffer” salts used, HEPES or dihydrogenphosphate, do not act as buffers since pH = 4.3 is too far away from the corresponding p*K*_a_ values of the two salts. Although all previous investigations with HRPC and PADPA were carried out with a sodium dihydrogenphosphate solution of pH = 4.3 (0.1 M), in the current work with heme *b*, a pH = 4.3 solution of 0.1 M HEPES was mainly used. The reason for this is the known positive effect HEPES molecules have on the activity of ferric heme *b*.^54^

## 2. Materials and methods

### 2.1. Commercial materials

HEPES (4-(2-hydroxyethyl)-1-piperazineethanesulfonic acid), 99%, for biochemistry, *M*_w_ = 238.30 g mol^−1^, lot A0233527, and hydrogen peroxide (H_2_O_2_) for analysis, 35 wt% solution in water, stabilized, lot: A0352305 were from Acros Organics. Sodium phosphate monobasic (sodium dihydrogenphosphate, NaH_2_PO_4_), ReagentPlus, ≥99.0%, lot BCBQ6142V, was purchased from Sigma-Aldrich. PADPA (*p*-aminodiphenylamine, *N*-phenyl-*p*-phenylenediamine), 98%, *M*_w_ = 184.24 g mol^−1^, lot MKBX9690V was from Aldrich Chemistry. PADPA was recrystallized from *n*-hexane as described before.^52^ Hemin from porcine, BioXtra, ≥97.0% (HPLC), lot BCCB6735, *M*_w_ = 651.94 g mol^−1^, was from Sigma. SDBS (dodecylbenzenesulfonic acid sodium salt, hard type), >95% (T), *M*_w_ = 348.48 g mol^−1^, was purchased from TCI Chemicals. Dimethyl sulfoxide (DMSO), Analytical reagent, lot 18K084026, was from VWR Chemicals, and *n*-hexane, for liquid chromatography, LiChrosolv, was from Merck. HRPC (horseradish peroxidase isoenzyme C), PEO-131, grade I, 271 U mg^−1^, RZ ≥ 3, lot 8153665000 was from Toyobo Enzymes. TMB (3,3′,5,5′-tetramethylbenzidine), lot BCBV1333 was purchased from Aldrich Chemistry. Polypropylene Eppendorf tubes (2 mL reaction tubes) were purchased from Greiner Bio-One GmbH.

### 2.2. Preparation of stock solutions

#### 2.2.1. Aqueous pH 4.3 HEPES or phosphate salt solution

Two different salt solutions of pH = 4.3 were prepared in glass bottles, either by using HEPES salt or NaH_2_PO_4_. The salts were dissolved in Milli-Q water to yield in both cases 0.1 M. The pH was adjusted to pH = 4.3 by using 2 M HCl. Depending on the type of experiment, one of the two pH 4.3 salt solutions was used for preparing the reaction mixture and also for the preparation of micellar solutions of SDBS.

#### 2.2.2. SDBS stock solutions

Stock solutions of SDBS (40.0 mM) were prepared by dissolving 55.8 mg of SDBS in 4 mL of one of the two aqueous pH 4.3 solutions. After preparation, the solutions were stored at room temperature (RT, *T* ≈ 25 °C) and used within 2 weeks.

#### 2.2.3. HRPC stock solutions

Stock solutions of HRPC were prepared by dissolving ≈4.0 mg of HRPC in 1 mL of 0.1 M sodium phosphate buffer solution (pH = 7.0), yielding a HRPC concentration of ≈70 μM. The exact concentration was determined spectrophotometrically by using as molar absorption *ε*_403_ = 102 000 M^−1^ cm^−1^ which gave a concentration of 77.6 μM.^16^ Such stock solution was kept at *T* = 4 °C. It remained very stable and could be used for at least for up to 2 years after preparation. For carrying out the reactions, stock solutions of lower HRPC concentration (0.8 μM) were freshly prepared before use. This was done by adding 10 μL of the 77.6 μM HRPC stock solution to 990 μL of the appropriate aqueous pH 4.3 solution.

#### 2.2.4. Hemin stock solution

A hemin stock solution (6.0 mM) was prepared by dissolving 4.17 mg of hemin powder in 1069 μL of DMSO. This solution was freshly prepared (used within a day), due to a previous report on the adsorption of hemin onto plasticware.^56^

#### 2.2.5. PADPA stock solution

A stock solution of PADPA (150 mM) was prepared in Eppendorf tubes by dissolving 13.8 mg of recrystallized PADPA in 500 μL of DMSO. This solution was freshly prepared before use.

#### 2.2.6. H_2_O_2_ stock solution

A hydrogen peroxide stock solution (200 mM) was freshly prepared in Eppendorf tubes before use by dissolving 19.3 μL of 35 wt% hydrogen peroxide solution (≈10.4 M) in 980.7 μL of Milli-Q water.

### 2.3. Preparation and analysis of reaction mixtures

#### 2.3.1. Reaction mixture preparation

Unless otherwise stated, all reactions were carried out in 2 mL Eppendorf tubes at RT. If not mentioned else, the reaction time was always 24 h, and the final volume of all reactions was 1 mL. In cases where hemin was used as catalyst, the order of addition of the different stock solutions was the following: (1) aqueous pH 4.3 solution containing either 0.1 M HEPES or 0.1 M phosphate salt, (2) SDBS stock solution, (3) hemin stock solution, (4) PADPA stock solution, and (5) H_2_O_2_ stock solution. The initial concentrations in the reaction mixture were: 5.0 mM SDBS, 10 μM hemin, 1.0 mM PADPA, and 1.0 mM H_2_O_2_. In order to avoid any effects of DMSO on the physical state of hemin,^56^ the total DMSO content was kept below 1 vol%. When HRPC was used as catalyst, the order of addition of the stock solutions was the following: (1) aqueous pH 4.3 solution containing 0.1 M HEPES or 0.1 M phosphate salt, (2) SDBS stock solution, (3) PADPA stock solution, (4) HRPC stock solution, and (5) H_2_O_2_ stock solution. The initial concentrations in the reaction mixture were: 3.0 mM SDBS, 30 nM HRPC, 1.0 mM PADPA, and 1.0 mM H_2_O_2_. In both systems, after the addition of each component, the reaction was gently mixed by flipping the Eppendorf tube with a closed cap up and down. When controls were performed, the order of addition of the stock solutions stayed the same, just one of the stock solutions was not used but replaced by an appropriate amount of the aqueous pH 4.3 solution.

#### 2.3.2. UV/vis/NIR, EPR and Raman spectroscopy measurements

UV/vis/NIR: for all reactions which were carried out in 2 mL Eppendorf tubes and analyzed after a reaction time of 24 h, a volume of 350 μL was withdrawn from the reaction mixture and poured into a quartz cuvette (0.1 cm path length), and the UV/vis/NIR absorption spectrum was recorded with a JASCO V-670 spectrophotometer. Time-dependent changes of the absorption spectrum during the course of the reaction were carried out by running the reaction inside the quartz cuvette (*in situ* measurements). For this, the reaction mixture was first prepared in an Eppendorf tube, followed by immediate removal of a volume of 350 μL from the reaction mixture and placing it into a quartz cuvette (0.1 cm path length). The absorption spectrum was then recorded every 15 min (up to 24 h). EPR: three identical reaction mixtures were prepared in 2 mL Eppendorf tubes. The tubes were stored at RT. After a predetermined time, the entire reaction mixture of one of the three tubes was poured into a flat quartz cell, and the EPR spectrum was measured with a Bruker EMX X-band spectrometer. Raman: Raman spectra of the reaction mixtures were recorded with a DXR Raman microscope (Thermo Scientific). A HeNe gas laser with an excitation wavelength of 633 nm was used for all measurements. The laser power on the sample was kept at 4.0 mW. The reaction mixtures were prepared in 2 mL Eppendorf tubes and stored at RT. At predetermined times, 10 μL aliquots of the reaction mixture were transferred into the sample wells of the sample platform (Gold EZ-Spot Micro Mount sample slide, Thermo Scientific). After filling the well with the sample of the reaction mixture, the slide with the sample was placed on an *X*–*Y* motorized sample stage, and the laser beam was focused on the sample at an objective magnification of 10×. The scattered light was analyzed by the spectrograph with a 600 lines mm^−1^ grating. The exposure time was 10 s, and 10 exposures per spectrum were taken. Automatic fluorescence correction was performed using the OMNIC software.

## 3. Results and discussion

### 3.1. Determination of the “optimal reaction conditions” for poly(PADPA) formation with hemin as catalyst

With “optimal reaction conditions” we mean reaction conditions (i) at which the formed amount of favorable, dark-green, PANI-ES-like poly(PADPA) products is as high as possible, while (ii) the amount of unfavorable products containing branches and/or phenazine-type units is low. Moreover, (iii) the amount of products obtained from PADPA as monomer should allow UV/vis/NIR absorption measurements inside cuvettes with a path length of 0.1 cm without dilution of the reaction mixture; and (iv) precipitation of intermediates and products during the reaction or after completion of the reaction should not occur. These requirements were set by ourselves for practical reasons since they allow a clear and simple screening of different reaction conditions by direct UV/vis/NIR measurements without the involvement of possible effects originating from post-reaction treatments and from reaction product isolation. Our main interest was in finding reaction conditions that resulted in the appearance of absorption bands at *λ* ≈ 1000 and 420 nm (as expected for PANI-ES-like products) and absence of strong bands at *λ* ≈ 500–600 nm (*i.e.*, absence of undesired branching and/or phenazine-type structure formation), see Introduction. Based on our previous work on the enzymatic oxidative oligomerization of PADPA,^5^ the initial concentrations for the reactions with hemin were kept at [PADPA]_0_ = 1.0 mM and [H_2_O_2_]_0_ = 1.0 mM. Furthermore, based on previous literature reports on the catalytic activity of hemin, the concentration of hemin used in all measurements was kept constant at [hemin] = 10 μM.^57–59^ This concentration is also useful for direct spectrophotometric measurements of hemin itself in the region of the Soret band (A_400_ (*l* = 1 cm) ≈ 0.5–1.2), so that information about possible changes in the aggregation state of hemin upon changing the reaction medium (in the absence of PADPA, *i.e.*, without reaction) can be obtained.^17^

For reference reactions with HRPC, the PADPA and H_2_O_2_ concentrations used were the same as in the case of the reactions with hemin, [PADPA]_0_ = [H_2_O_2_]_0_ = 1.0 mM, and the HRPC concentration applied was the one applied in our previous work with AOT vesicles as templates:^5^ [HRPC] = 30 nM.

As mentioned in the Introduction, SDBS micelles were used for hosting hemin and as templates for the reaction. The determination of the optimal SDBS concentration was carried out separately for the hemin- and the HRPC-catalyzed reactions. For both optimizations, an aqueous 0.1 M HEPES solution with pH = 4.3 was used. The spectra measured after a reaction time of *t* = 24 h in the presence of different SDBS concentrations are shown in Fig. S-1 and S-2, ESI.† The optimal SDBS concentration for the hemin system was found to be 5.0 mM, while for the HRPC system it is 3.0 mM. The same optimal SDBS concentrations were also obtained when an aqueous 0.1 M dihydrogenphosphate solution at pH = 4.3 was used instead of the HEPES solution (data not shown). For both systems, SDBS may play at least two roles, see Introduction. First, hemin (amphiphilic and positively charged at low pH if water molecules coordinate at the 5^th^ and 6^th^ coordination site) and HRPC (overall positively charged at pH = 4.3, pI(HRPC) = 8.8,^21^ are expected to bind to SDBS micelles. Therefore, the micelles localize the catalytic steps of the reaction. Second, cationic PADPA and (some of) the reaction intermediates and products are expected to bind to SDBS micelles, similarly to what was shown in the case of anionic AOT vesicles.^11,52^ Such binding results in increased substrate concentration in the area of the micelles if compared to the concentration in bulk solution. At optimal reaction conditions, no matter whether hemin or HRPC was used, the reaction products remained dispersed in the aqueous solution for several days without visible precipitation. This supports the existence of product-SDBS micelle interactions, with SDBS acting as counterion (dopant) of the formed PANI-ES-like products with their positively charged backbone. Moreover, in the presence of SDBS micelles, hemin at 10 μM is soluble in 0.1 M HEPES (or 0.1 M H_2_PO_4_^−^) at pH = 4.3, while without SDBS, at the same hemin concentration aggregates form (small particles that are visible by the naked eye), see Fig. S-3.†

### 3.2. Comparison of the reaction products obtained with hemin or HRPC as catalyst

#### 3.2.1. UV/vis/NIR spectroscopy measurements

**Fig. 2:**
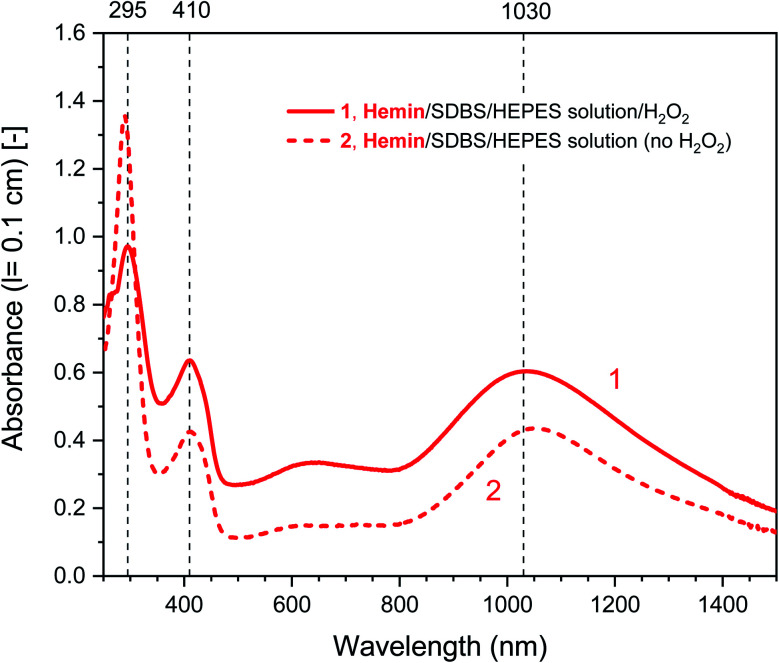
UV/vis/NIR absorption spectra of reaction mixtures prepared in HEPES solution containing hemin as catalyst, PADPA as monomer, and SDBS micelles, either with added H_2_O_2_ (1, solid line), or without added H_2_O_2_ (2, dashed line), recorded after a reaction time *t* = 24 h at RT. Reaction conditions: 0.1 M HEPES, pH = 4.3; [hemin] = 10 μM; [PADPA]_0_ = 1.0 mM; [SDBS] = 5.0 mM; [H_2_O_2_]_0_ = 1.0 mM (for 1). The contribution of hemin to A_400_ (*l* = 0.1 cm) is ≈ 0.066 or less; and to A_500_ and A_610_ (*l* = 0.1 cm) ≈ 0.012 and ≈ 0.005, respectively, see Fig. S-3.†

**Fig. 3:**
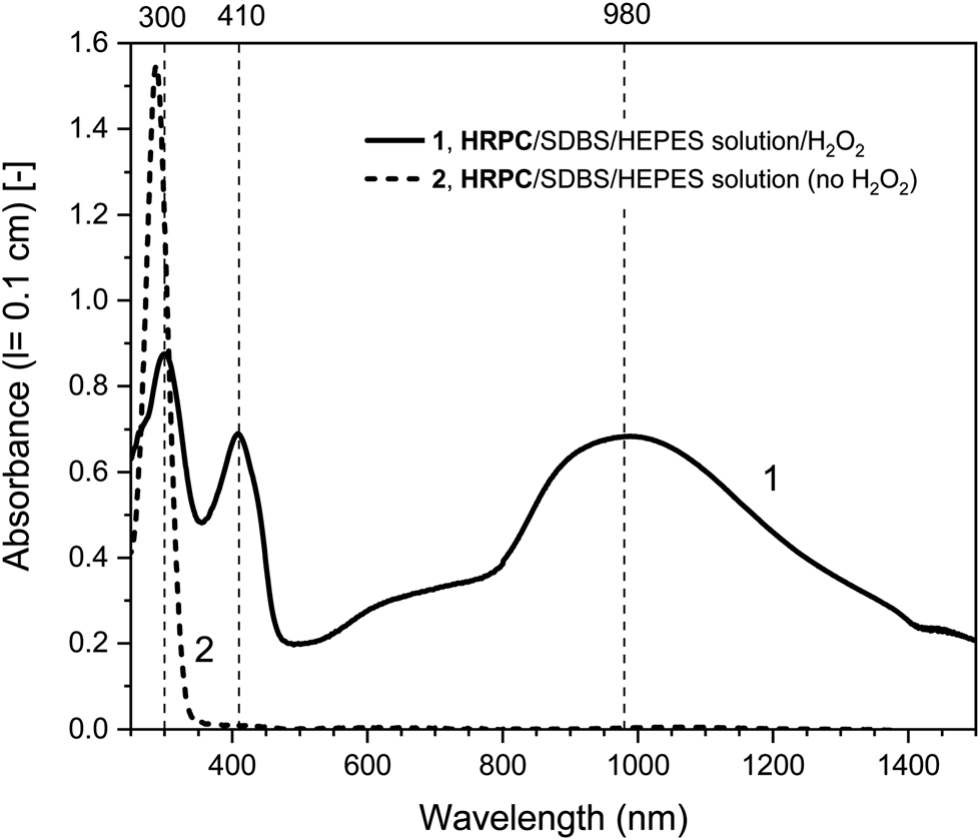
UV/vis/NIR absorption spectra of reaction mixtures prepared in HEPES solution containing HRPC as catalyst, PADPA as monomer, and SDBS micelles, either with added H_2_O_2_ (1, solid line), or without added H_2_O_2_ (2, dashed line), recorded after a reaction time *t* = 24 h at RT. Reaction conditions: 0.1 M HEPES, pH = 4.3; [HRPC] = 30 nM; [PADPA]_0_ = 1.0 mM; [SDBS] = 3.0 mM; [H_2_O_2_]_0_ = 1.0 mM (for 1).

For the determined “optimal reaction conditions”, both with hemin or HRPC as catalyst, the reaction mixtures were analyzed and compared with each other by recording UV/vis/NIR absorption spectra after running the reaction for *t* = 24 h at RT (see Fig. 2 and 3, solid lines). For both systems, the general features of the spectra are very similar. Absorption bands at *λ*_max_ ≈ 1000 and ≈ 410 nm are indications for the presence of favorable PANI-ES-type products (see Introduction), although one has to be aware of the fact that in the case of hemin (10 μM), the Soret band absorption of the heme group contributes to A_400_ as well (see the legend of Fig. 2 and S-3†). The band at *λ* ≈ 615 nm most likely indicates the presence of undesired phenazine-like units.^5^ The band at *λ* ≈ 300 nm originates in part from remaining PADPA. The intensity of this band is very high at the beginning of the reaction and decreases during the course of the reaction when PADPA is consumed (see below). Based on A_300_ (as well as A_≈1000_) measured after *t* = 24 h, it seems that the PADPA conversion was a bit higher for the HRPC system as compared to the hemin system. The ratio of absorption intensities at *λ* ≈ 1000 and 610 nm was 1.8 for the hemin system (A_1030_/A_610_) and 2.4 for the HRPC system (A_980_/A_610_). This qualitative resemblance of the two UV/vis/NIR spectra of the reaction mixture after *t* = 24 h is an indication that the products obtained from the two systems are similar but not identical. A higher value of *λ*_max_ for the π → polaron transition band, 1030 nm for the hemin system compared to 980 nm for the HRPC system, indicates higher polaron delocalization and higher electrical conductivity of the poly(PADPA) products synthesized with hemin. The spectrum of the oligo- or poly(PADPA) products obtained with HRPC as catalyst were also compared with the spectrum of the products obtained from PADPA in our previous investigations, again with HRPC as catalyst and H_2_O_2_ as terminal oxidant, but in the presence of AOT vesicles, again at pH = 4.3 in 0.1 M H_2_PO_4_^−^ solution, also with [PADPA]_0_ = [H_2_O_2_]_0_ = 1.0 mM and [HRPC] = 30 nM, see Fig. S-4.†^5^ In both cases, there is a clear absorption band at *λ*_max_ = 410 nm (with basically the same intensity), and there are strong absorptions in the NIR region of the spectrum, with *λ*_max_ = 1097 nm for the reaction run in the presence of AOT vesicles,^5^ and *λ*_max_ = 980 nm for the reaction in the presence of SDBS micelles (this work). In both cases, there is absorption in the region of *λ* = 500–600 nm with differences in absorption band positions (Fig. S-4†). Although we are not able to unambiguously explain the observed differences when using SDBS micelles *vs.* AOT vesicles as templates, a dependence of the absorption in the vis/NIR region of the absorption spectrum on the type of template used was also seen before in the case of the oxidative oligomerization of PADPA with *Trametes versicolor* laccase and O_2_ for reactions carried out at pH = 3.5.^60^ Such differences most likely reflect not only differences in the molecular constitution (*i.e.*, the type of bonds formed between structural units), but also differences in (i) the degree of protonation of the nitrogen atoms present in the products, (ii) the oxidation state, (iii) the electron conjugation length, and/or (iv) molecular conformations.

Further comparative measurements were carried out for the hemin- and the HRPC-catalyzed reaction in the presence of 0.1 M dihydrogenphosphate instead of 0.1 M HEPES, again at pH = 4.3. The UV/vis/NIR absorption spectra of the reaction mixtures prepared with the H_2_PO_4_^−^ solution and incubated for *t* = 24 h at RT are shown in Fig. S-5† together with the spectra obtained in the presence of the HEPES solution (data of Fig. 2 and 3). This comparison was made due to a previous report of Travascio and coworkers,^54^ in which it was demonstrated that the catalytic activity of a DNA aptamer–hemin complex in the presence of Triton X-100 may depend significantly on the salt type used, and that the use of HEPES at pH = 8.0 may result in a more efficient reaction as compared to phosphate (analyzed with ABTS^2−^, 2,2′-azino-bis(3-ethylbenzothiazoline-6-sulfonate), as substrate). Our aim was to find out whether a similar salt-type dependence of the activity of hemin also exists in the case of PADPA as substrate in the presence of SDBS micelles. The measurements showed that this is indeed clearly the case (Fig. S-5†). Although there was no big difference in the absorption peak positions for the two systems (at *λ* ≈ 1030–1080, 400 nm, and 615 nm), two observations are obvious: (i) A_≈1000_ and A_≈400_ after *t* = 24 h were much higher for the reaction mixture prepared with the HEPES solution, while A_≈500–600_ was in both cases about the same; (ii) A_≈300_ after *t* = 24 h remained at a higher value for the reaction in the presence of dihydrogenphosphate as compared to the HEPES system (Fig. S-5†). These two observations clearly indicate higher conversion and a better “product quality” (higher A_≈1000_/A_500_ and A_≈1000_/A_600_ ratios) if the HEPES solution is used instead of the dihydrogenphosphate solution. For the reactions with HRPC, the band positions and intensities in the vis and NIR region of the absorption spectrum were very similar, although the spectrum recorded after *t* = 24 h at RT for the reaction run in the HEPES solution had slightly higher intensities at *λ* ≈ 400 nm and at the band maximum in the NIR region. However, the band maximum position and intensity were different (*λ*_max_ = 980 nm, with A_980_ (0.1 cm) = 0.68, for the HEPES solution and *λ*_max_ = 1074 nm, with A_1074_ (0.1 cm) = 0.63, for the dihydrogenphosphate solution) (Fig. S-5†). These latter intensities are similar to the value determined previously with AOT vesicles as templates (0.1 M NaH_2_PO_4_)^5^ instead of SDBS micelles with the same [PADPA]_0_, [H_2_O_2_]_0_, and [HRPC] (Fig. S-4,† A_1100_ (0.1 cm) ≈0.68 after *t* = 24 at RT).

As an important result from this HEPES *vs.* dihydrogenphosphate comparison is that under the conditions used the presence of HEPES has a positive effect on the outcome of the hemin-catalyzed formation of poly(PADPA). Whether this positive “HEPES effect” is a direct consequence of the influence the HEPES molecules have on the catalytic activity of hemin, or whether it is an effect which originates from HEPES-substrate interactions, was investigated by using TMB (3,3′,5,5′-tetramethylbenzidine) as substrate ([TMB]_0_ = [H_2_O_2_]_0_ = 0.3 mM, pH = 4.3, see Fig. S-6†). The initial rate of TMB oxidation was about 3.6 times faster in the presence of HEPES as compared to dihydrogenphosphate. This supports a direct effect of HEPES on the catalytic activity of hemin, possibly by coordinating to the iron atom at the 5^th^ coordination site. For the results presented in the following, the reaction mixtures usually contained 0.1 M HEPES at pH = 4.3 (optimal conditions). Only for comparative measurements, a 0.1 M H_2_PO_4_^−^ solution at pH = 4.3 was used as well.

Different control measurements were carried out for both the hemin- and the HRPC-catalyzed reactions in the presence of 0.1 M HEPES and PADPA (1.0 mM) at pH 4.3, whereby one of the other components present in the optimal reaction mixtures was omitted, either SDBS, catalyst (hemin or HRPC), or hydrogen peroxide. In the case of the HRPC system, the reaction mixture did not turn green after *t* = 24 h at RT if one of the mentioned components was absent (no absorption in the near-infrared region of the spectrum, *i.e.*, no formation of PANI-ES-like products), see Fig. S-7.† Without H_2_O_2_ (but with HRPC and SDBS), no reaction occurred (the spectrum measured after *t* = 24 h was the spectrum of PADPA (Fig. 3, dashed line); without HRPC (but with H_2_O_2_ and SDBS), the absorption at *λ* ≈ 300 nm due to PADPA remained high after *t* = 24 h (no significant PADPA conversion); without SDBS (but with HRPC and H_2_O_2_), a reaction occurred but most of the brown products that formed precipitated, as already observed before.^5^ Therefore, the oxidation and oligomerization or polymerization of PADPA into PANI-ES-like oligo- or poly(PADPA) products with HRPC requires the presence of H_2_O_2_ as well as SDBS (Fig. S-7†). In the case of the hemin system, the situation is different. For two control measurements, the outcome was as expected: (i) in the absence of hemin (with H_2_O_2_ and SDBS), or (ii) in the absence of SDBS (with hemin and H_2_O_2_), no significant amounts of the green PANI-ES-like products were obtained (no significant absorption at *λ* ≈ 1000 nm, presence of a weak absorption centered around *λ* = 600 nm) (Fig. S-8,† compare also with the spectrum shown in Fig. S-1,† for “no SDBS”). More interesting and somewhat surprising, however, was the outcome of the control measurements without H_2_O_2_ (but with hemin and SDBS). The reaction proceeded quite well without added H_2_O_2_, resulting after *t* = 24 h at RT in an absorption spectrum with *λ*_max_ = 1051 nm with A_1051_ (0.1 cm) = 0.44 and *λ*_max_ = 412 nm with A_412_ (0.1 cm) = 0.43, as compared to *λ*_max_ = 1030 nm with A_1030_ (0.1 cm) = 0.60 and *λ*_max_ = 410 nm with A_410_ (0.1 cm) = 0.64, for the reaction run under the “optimal conditions”, *i.e.*, in the presence of H_2_O_2_, hemin and SDBS (Fig. 2, dashed line and Fig. S-8†). This observed reactivity of hemin without H_2_O_2_ indicates not only (i) how insightful control measurements can be, but also (ii) that – under the conditions used (0.1 M HEPES, pH = 4.3) – the oxidation of PADPA with hemin in the absence of added H_2_O_2_ cannot proceed *via* a peroxidase-like mechanism (see Scheme S-1†), where H_2_O_2_ first oxidizes the heme group in a two-electron oxidation reaction to yield compound I, which then initiates the oxidation of the substrate (PADPA) in two consecutive one-electron oxidation reactions. Compound I formation – if compound I forms at all – must occur in a different way in the absence of H_2_O_2_. For the hemin-catalyzed reaction without added H_2_O_2_, the absorption at *λ* ≈ 600 nm after *t* = 24 h at RT was lower than in the case of the reaction run under the “optimal conditions” (with H_2_O_2_) (Fig. 2). The ratio of A_1051_/A_600_ for the reaction mixture where no H_2_O_2_ was added was 3.0, as compared to A_1030_/A_600_ = 1.9 for the reaction run under “optimal conditions” (with H_2_O_2_). Understanding how the hemin-catalyzed oxidation of PADPA in the absence of H_2_O_2_ occurs is an interesting challenge for detailed future investigations (see below).

For a direct comparison, Fig. S-9† shows the UV/vis/NIR absorption spectra of the reaction mixtures containing either hemin or HRPC and incubated for *t* = 24 h at RT (in the HEPES solution with or without H_2_O_2_).

The same set of control measurements for the hemin- and HRPC-catalyzed reactions were also carried out in the presence of aqueous 0.1 M dihydrogenphosphate solution instead of 0.1 M HEPES at pH 4.3 (see Fig. S-10 and S-11†). Qualitatively, similar results were obtained when using the dihydrogenphosphate solution as compared to using the HEPES solution: For HRPC, there was no significant reaction without H_2_O_2_ (Fig. S-11†); for hemin, a clear reaction also occurred without H_2_O_2_. Moreover, the absorption spectrum of the oligo- or poly(PADPA) products obtained after *t* = 24 h at RT was about the same for the reaction run in the H_2_PO_4_^−^ solution with H_2_O_2_ as compared to without H_2_O_2_ (Fig. S-10†). Obviously, the addition of H_2_O_2_ had no beneficial effect at all if the reaction was run in the dihydrogenphosphate solution at pH = 4.3. This is different to what we observed with the HEPES solution, where the added H_2_O_2_ still was advantageous (see Fig. S-5†): compared to the reactions run in the dihydrogenphosphate solution with hemin and PADPA, the reactions in HEPES solution were more efficient (clearly higher intensity in the NIR region of the absorption spectrum of the products obtained).

Using TMB as substrate and measuring the initial rate of TMB oxidation in the presence of hemin, qualitatively the same results were obtained as with PADPA, see Fig. S-12 and S-13:† (i) with the HEPES solution, TMB was also oxidized without added H_2_O_2_, but the TMB oxidation was much faster with added H_2_O_2_ (Fig. S-12†); (ii) with the dihydrogenphosphate solution, the same initial rate of TMB oxidation was measured, independent on whether H_2_O_2_ was added or not (Fig. S-13, ESI†); and (iii) with the HEPES solution, the initial rate of TMB oxidation in the presence of H_2_O_2_ was ≈3.6 times faster than with the H_2_PO_4_^−^ solution (please compare Fig. S-12 and S-13†).

Overall, the “best” conditions for oxidizing PADPA to PANI-ES-like oligo- or poly(PADPA) products with hemin is to use the HEPES solution with added H_2_O_2_ at the optimal reaction conditions already mentioned above: 0.1 M HEPES, pH = 4.3; [SDBS] = 5.0 mM; [hemin] = 10 μM, [PADPA]_0_ = 1.0 mM; [H_2_O_2_]_0_ = 1.0 mM.

**Fig. 4:**
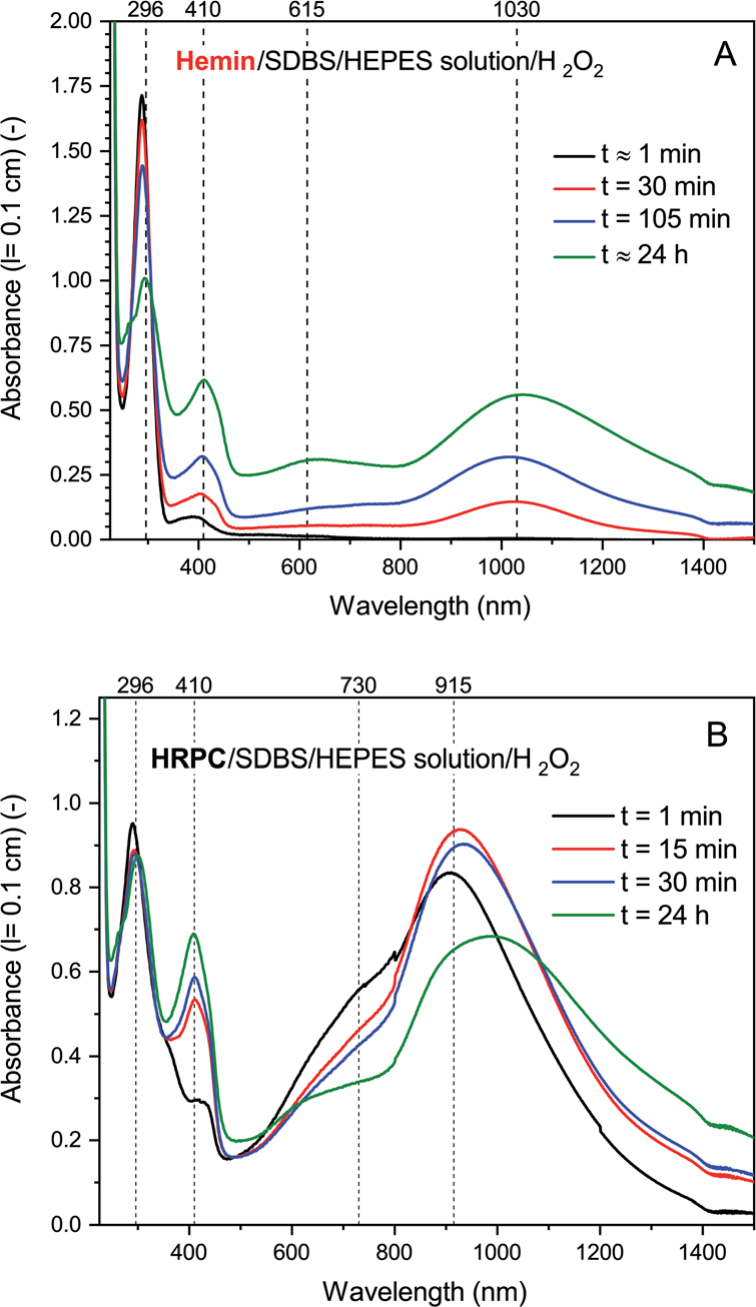
UV/vis/NIR absorption measurements during the reaction of PADPA with either hemin (A) or HRPC (B) as catalyst and added H_2_O_2_. Reaction conditions: (A) 0.1 M HEPES pH 4.3 solution, [SDBS] = 5.0 mM, [hemin] = 10.0 μM, [PADPA]_0_ = 1.0 mM, [H_2_O_2_]_0_ = 1.0 mM; (B) 0.1 M HEPES pH 4.3 solution, [HRP] = 30.0 nM, [SDBS] = 3.0 mM, [PADPA]_0_ = 1.0 mM [H_2_O_2_]_0_ = 1.0 mM. For the sake of clarity, only 4 measurements (the most prominent ones) per system are shown; a complete data set can be found in Fig. S-14 and S-15.† Total reaction time *t* ≈ 24 h, RT.

In Fig. 4, the time-dependent changes of the UV/vis/NIR absorption spectrum for the hemin- and the HRP-catalyzed oxidative oligomerization of PADPA in the presence of SDBS micelles and with added H_2_O_2_ in the HEPES solution are shown.

With hemin, the development of the UV/vis/NIR absorption spectrum over time is relatively simple. There is an increase of absorption above *λ* ≈ 380 nm over time with a clear appearance of bands centered around *λ* ≈ 1030 and ≈ 410 nm (indication of the formation of favorable PANI-ES-type products). A broad band in the region of *λ* ≈ 615 nm is also clearly developing, which indicates formation of unfavorable products containing phenazine-type units. There is also a decrease of absorbance at *λ* ≈ 300 nm, which is an indication of PADPA being consumed during the reaction. The rate of reaction but not the pattern of the spectral evolution was affected by the type of salt present (HEPES *vs.* dihydrogenphosphate) and the presence or absence of H_2_O_2_ (see Fig. S-16–S-18†). The fastest initial rate of spectral development in the hemin-catalyzed system was observed when the HEPES salt and H_2_O_2_ were used, *i.e.*, the “optimal reaction conditions”.

With HRPC, the development of the UV/vis/NIR absorption spectrum with time was rather different from the spectral development of the reaction mixtures containing hemin. During the first 30 min, there was a sharp increase in absorbance of the bands positioned at *λ* ≈ 410 and ≈ 915 nm, which indicates the presence of PANI-ES-type products. In addition, there was a decrease in absorbance at A_296_, which shows that PADPA was consumed during the reaction. After 30 min, there was a slow decrease of A_915_ and at the same time an increase of absorption intensity in the NIR region, at *λ* = 1200–1400 nm, with an isosbestic point at *λ* ≈ 1100 nm. In addition, during the reaction, a shoulder at *λ* ≈ 700 nm was always present. Please note that the spectrum obtained after *t* = 24 h was slightly different from the one obtained for the same reaction conditions shown in Fig. 2. This difference originates from the different reaction containers used in the two cases (see Materials and methods).

**Fig. 5:**
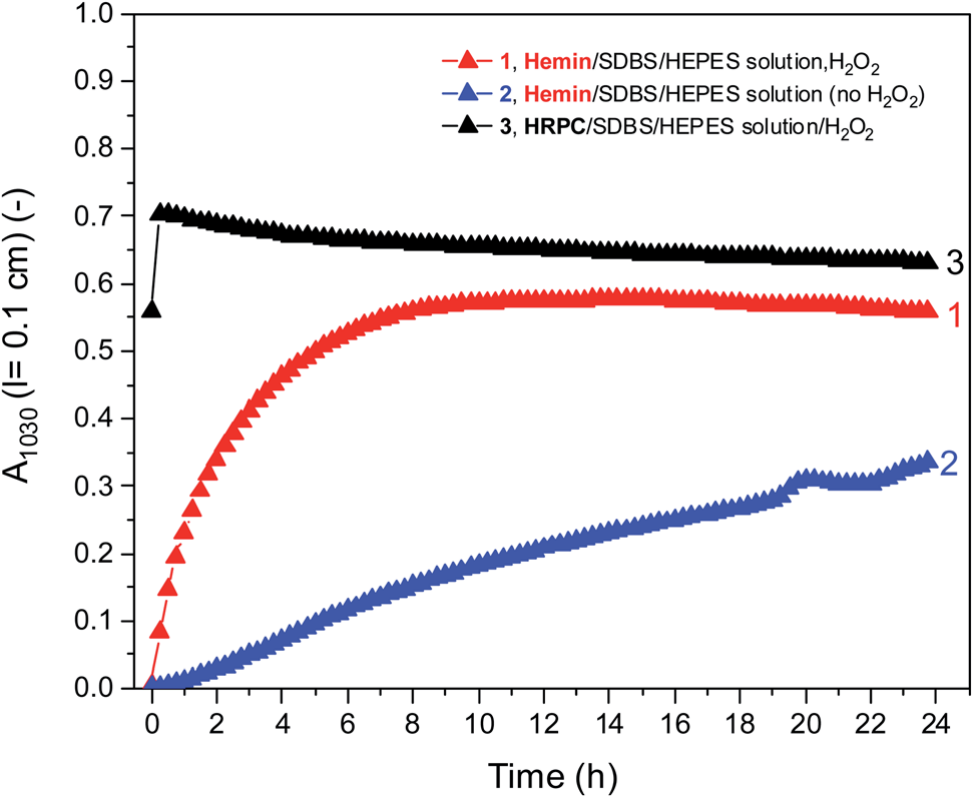
Comparison of the changes of A_1030_ during the reaction of PADPA in 0.1 M HEPES solution (pH = 4.3) with either hemin or HRPC as catalyst at RT. Reaction conditions: (1) [hemin] = 10.0 μM, [SDBS] = 5 mM, [PADPA]_0_ = 1.0 mM, [H_2_O_2_]_0_ = 1.0 mM; (2) [hemin] = 10.0 μM, [SDBS] = 5 mM, [PADPA]_0_ = 1.0 mM, no H_2_O_2_]; (3) [HRPC] = 30 nM, [SDBS] = 3 mM, [PADPA]_0_ = 1.0 mM, [H_2_O_2_]_0_ = 1.0 mM. Data points were obtained by running the reaction inside quartz cuvettes with a pathlength of *l* = 0.1 cm and recording the spectrum every 15 min for up to *t* ≈ 24 h at RT.

Fig. 5 shows a direct comparison of the time-dependent changes of A_1030_ for the reaction mixtures prepared with the HEPES solution with hemin as catalyst, with (1) or without (2) H_2_O_2_. Data for the HRPC system with H_2_O_2_ (3) are also shown (note that without H_2_O_2_ no reaction occurred, see Fig. 3). In the case of the HRPC-catalyzed reaction, A_1030_ leveled off after *t* ≈ 15 min already, with a continuous slow decrease beyond this time due to a broadening of the spectrum into the NIR region (see Fig. 4B). For the hemin-catalyzed system, a plateau value was reached after *t* ≈ 8 hours in the presence of H_2_O_2_, while without added H_2_O_2_, the reaction clearly was much slower but A_1030_ kept gradually increasing (at least up to *t* = 24 h). This latter comparison is again a clear proof that for the hemin-catalyzed reaction the addition of H_2_O_2_ is not necessary for the reaction to proceed (see Fig. 2), but that the rate of reaction with addition of H_2_O_2_ is much faster than without added H_2_O_2_.

For the sake of completeness, kinetic measurements were also carried out by using the 0.1 M H_2_PO_4_^−^ solution instead of the 0.1 M HEPES solution, see Fig. S-19.† These measurements confirmed the lower activity of hemin in the H_2_PO_4_^−^ solution, in which the addition of H_2_O_2_ had no beneficial effect at all (see also Fig. S-10†). For all further measurements by using EPR and Raman spectroscopy as analytical methods, the focus was on reaction mixtures prepared in 0.1 HEPES at pH = 4.3. Nevertheless, the results obtained by using 0.1 M H_2_PO_4_^−^ at pH = 4.3 are also mentioned, with the corresponding data shown in the ESI.

#### 3.2.2. EPR spectroscopy measurements

**Fig. 6:**
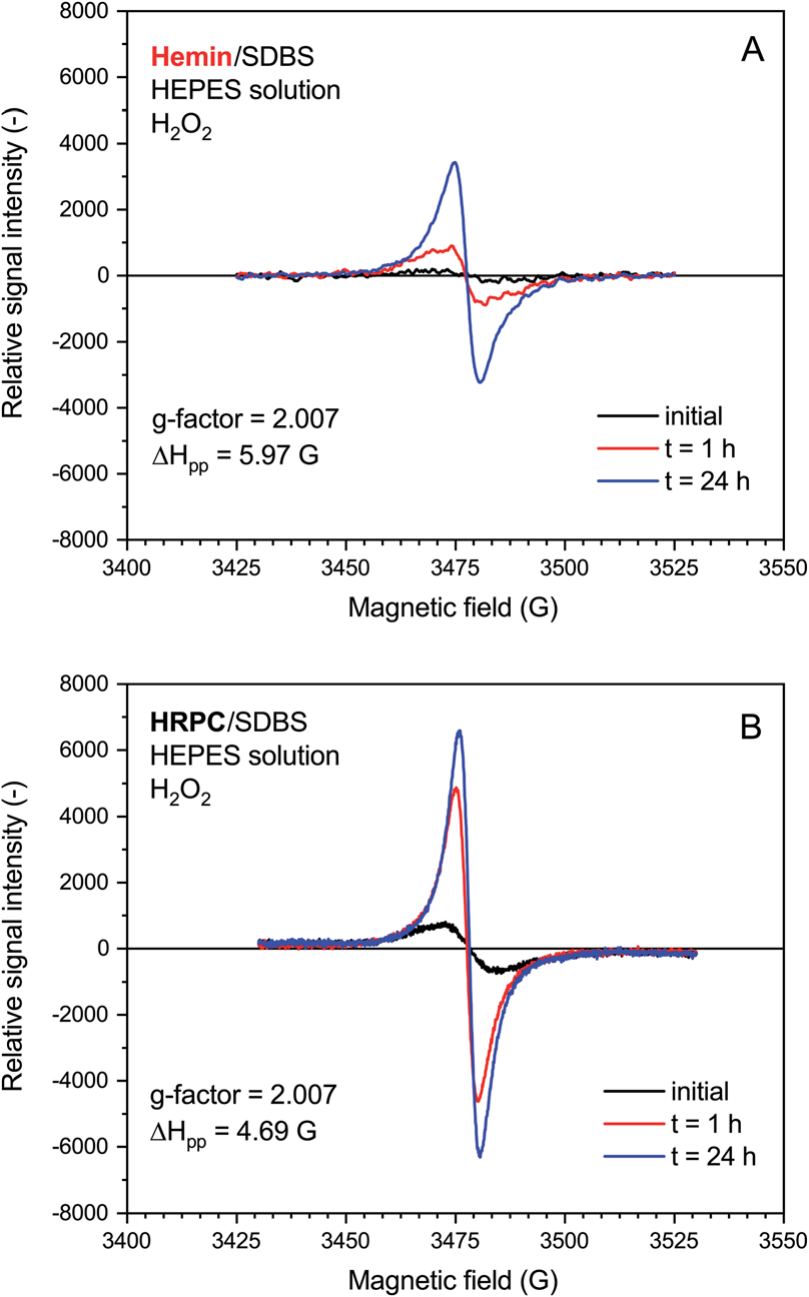
EPR spectroscopy measurements for reaction mixtures prepared with 0.1 M HEPES, pH = 4.3, with either hemin (A) or HRPC (B) as catalyst. Reaction conditions: (A) [hemin] = 10.0 μM, [SDBS] = 5.0 mM, [PADPA]_0_ = 1.0 mM, [H_2_O_2_]_0_ = 1.0 mM; (B) [HRPC] = 30.0 nM, [SDBS] = 3 mM, [PADPA]_0_ = 1.0 mM, [H_2_O_2_]_0_ = 1.0 mM. The reactions were run at RT inside Eppendorf reaction tubes and analyzed after *t* ≈ 2 min, 1 h or 24 h. *G*-factor and Δ*H*_pp_ values are calculated for the spectrum recorded at *t* = 24 h.

Under the elaborated optimal conditions for the hemin- and HRPC-catalyzed reactions (use of the HEPES solution), EPR measurements were carried out. In both cases, the presence of an EPR signal in the reaction mixtures that were prepared in Eppendorf reaction tubes and analyzed 1 h or 24 h after the start of the reactions was in agreement with the formation of the polaron form of PANI-ES type products (presence of unpaired electrons, with characteristic absorption at *λ*_max_ ≈ 420 and 800–1100 nm, see Introduction), see Fig. 6.

The rate of increase of the intensity of the EPR signal with time was slower if hemin was used as catalyst, as compared to HRPC. This is in agreement with the kinetic data obtained from the UV/vis/NIR absorption measurements, given that the presence of an absorption band in the NIR region of the spectrum is due to the presence of unpaired electrons that give rise to an EPR spectrum (see Introduction). The relative quantity of radicals produced till *t* = 24 h was significantly higher for the reaction run with HRPC as compared to hemin. The calculated *g*-factor values in both systems are identical (2.007, see Fig. 6) and are in good agreement with previously reported values for the emeraldine salt form of PANI.^61,62^ The EPR signal width (Δ*H*_pp_) for the spectrum recorded after *t* = 24 h indicates that the radical species are more uniform in the HRPC-catalyzed reaction (Δ*H*_pp_ = 4.69 G) than for the reaction run with hemin (Δ*H*_pp_ = 5.97 G), see Fig. 6.

**Fig. 7:**
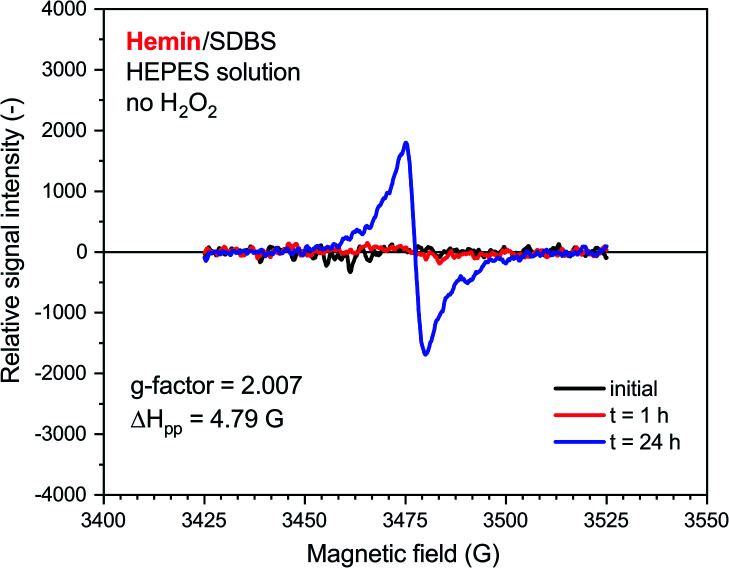
EPR spectroscopy measurements for a reaction mixture prepared with 0.1 M HEPES, pH = 4.3, with hemin without added H_2_O_2_. Reaction conditions: [hemin] = 10.0 μM, [SDBS] = 5.0 mM, [PADPA]_0_ = 1.0 mM, no H_2_O_2_. The reaction was run at RT inside an Eppendorf reaction tube and analyzed after *t* ≈ 2 min (initial), 1 h or 24 h. *G*-factor and Δ*H*_pp_ values are calculated for the spectrum recorded at *t* = 24 h.

A very weak EPR signal was also observed in the reaction mixture containing hemin without added H_2_O_2_, see Fig. 7. The relative intensity after *t* = 24 h was, however, much lower than for the reaction run with H_2_O_2_ (compare Fig. 7 with Fig. 6). This is again in qualitative agreement with the UV/vis/NIR absorption measurements, see Fig. 2. The *g*-factor determined for the spectrum recorded after *t* = 24 h is identical with the ones described in Fig. 6. Interestingly, Δ*H*_pp_ is more similar to the one determined for the spectrum of the HRPC system than for the spectrum of the hemin system with added H_2_O_2_.

EPR measurements were also carried out for reaction mixtures containing hemin in 0.1 M H_2_PO_4_^−^ solution (pH = 4.3) instead of 0.1 M HEPES (pH = 4.3), both in the presence and absence of H_2_O_2_, see Fig. S-20.† The data are again in good qualitative agreement with the UV/vis/NIR measurements, *i.e.* there was no beneficial effect of H_2_O_2_ on the EPR signal intensity for the reaction with hemin in the reaction mixture containing dihydrogenphosphate. Furthermore, when the reaction mixture was carried out in the 0.1 M dihydrogenphosphate solution (pH = 4.3) the relative intensity of the EPR signal obtained when HRPC was used as catalyst was much higher than when hemin was used.

#### 3.2.3. Raman spectroscopy measurements

For each of the aforementioned reaction mixtures using hemin or HRPC as catalyst under optimal reaction conditions, Raman spectroscopy measurements were carried out, again *in situ*, like in the case of the UV/vis/NIR and EPR measurements, *i.e.*, without isolation of the reaction products.

**Fig. 8:**
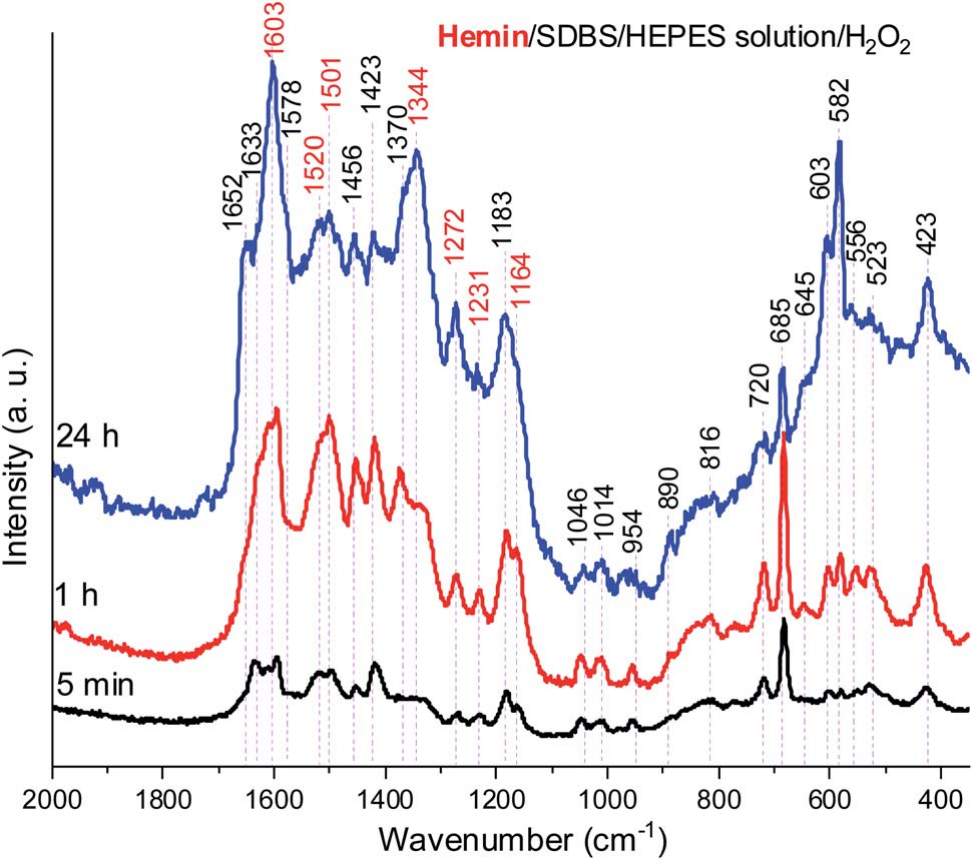
Raman spectra of a reaction mixture prepared with an aqueous 0.1 M HEPES solution at pH 4.3, with [hemin] = 10.0 μM, [SDBS] = 5 mM, [PADPA]_0_ = 1.0 mM, [H_2_O_2_]_0_ = 1.0 mM, recorded at *t* = 5 min, 1 h, and 24 h at RT. The bands characteristic for PANI-ES products are labeled in red, see text; a.u., arbitrary units.

In Fig. 8, Raman spectra obtained for the reaction mixture containing hemin are shown for reaction times *t* = 5 min, 1 h, and 24 h. For the reaction mixture analyzed after *t* = 5 min, the Raman spectrum contained numerous bands that originate from starting materials and early reaction intermediates. As time progressed, the spectrum changed, and several bands that are typical for PANI-ES-like oligomeric products of PADPA appeared, as discussed in the following.

A number of bands that are characteristic for structural units of ordinary (“standard”) PANI can be observed at *ν̃* = 1620 cm^−1^ (m, C–C stretching vibrations of benzenoid (B) rings, *ν*(C–C)_B_), 1603 cm^−1^ (m, C=C and C–C stretching vibrations of quinonoid (Q) and semiquinonoid (SQ) rings, *ν*(C=C)_Q_ and *ν*(C–C)_SQ_), 1520 cm^−1^ (m, N–H bending vibration, *δ*(N–H), commonly related to SQ (polaron) structures in PANI), 1501 cm^−1^ (m, C=N stretching in quinonediimine units, *ν*(C=N)_Q_), 1340 cm^−1^ (w, C–N˙^+^ stretching vibration in SQ (polaron) units, *ν*(C–N˙^+^)_SQ_, characteristic for the conducting form of PANI, the emeraldine salt PANI-ES), 1272 cm^−1^ (w, C–N stretching vibrations in B rings, *ν*(C–N)_B_˙__), 1231 cm^−1^ (w, *ν*(C–N)_B_), 1181 cm^−1^ (m, C–H bending in-plane vibration of B ring, *δ*(C–H)_B_), and 1164 cm^−1^ (m, C–H bending in-plane vibration of Q ring, *δ*(C–H)_Q_).^53^ The Raman spectrum also indicates formation of structural units different from those of “standard” PANI, by the bands observed at *ν̃* = 1633 cm^−1^ (m, attributable to C–C ring-stretching vibrations in phenazine-, *N*-phenylphenazine- and/or phenoxazine-type units, mixed with the C–C stretching vibration of the B ring, *ν*(C–C)_B_), 1578 cm^−1^ (sh, phenazine-type of units), 1456 cm^−1^ (w, assignable to C–C ring stretching/C=N stretching in quinonoid type of units that are probably present in short chains/branches and in substituted phenazine- and *N*-phenylphenazine-type segments), 1423 cm^−1^ (m, assignable to the ring-stretching vibration in phenazine-, *N*-phenylphenazine- and phenoxazine-type units, combined with a contribution from DMSO), 1370 cm^−1^ (vw, *ν*(C–N^+^) ring stretching vibration in substituted *N*-phenylphenazine-type units and to phenoxazine-type units/*ν̃*(C–N˙^+^)_SQ_ vibration in polaron-SQ structures of low delocalization), and 582 cm^−1^ w, a band at similar position in spectra of PANI has been attributed to substituted phenoxazine-like units.^53,63,64^ Such phenoxazine-like units could have been formed by the oxidative intramolecular cyclization of substituted *ortho*-aminophenol units, formed as products of hydrolysis of fully oxidized pernigraniline-like oligomers,^65^ and substituted phenazine- and *N*-phenylphenazine-type units.^53^ Contributions of C–S stretching vibration *ν*(C–S) and SO_2_ deformation vibration *δ*(SO_2_) probably originating from the anion of SDBS (DBS) to the band at 582 cm^−1^ are also possible.^66^ The weak band in the spectrum at *t* = 5 min observed at 603 cm^−1^ could be assigned to *δ*(SO_2_) vibrations, originating from SDBS,^66^ and benzenoid (B) ring deformation in PANI-like segments of oligo- or poly(PADPA).¶¶Sulfonates often have two bands in the range *ν̃* = 610–500 cm^−1^ due to SO_2_ deformation vibrations.^66^^53,67^ In the Raman spectrum recorded at *t* = 1 h, one can observe that the band at *ν̃* = 1370 cm^−1^, attributed to *ν*(C–N˙^+^)_SQ_ of polarons with lower delocalization/*N*-phenylphenazine and phenoxazine units, is well developed, being even stronger than the band at *ν̃* = 1340 cm^−1^ assigned to *ν*(C–N˙^+^)_SQ_ of more delocalized polarons. However, in the Raman spectrum for *t* = 24 h, the intensity ratio of these two bands is reversed, and the band at *ν̃* = 1370 cm^−1^ becomes a shoulder of the band at *ν̃* = 1344 cm^−1^.

The relative intensities of the two bands characteristic of conducting PANI-ES at *ν̃* = 1603 cm^−1^ and 1344 cm^−1^ increased as the reaction time increased, so that they are present as the most intense bands in the Raman spectrum recorded at *t* = 24 h, along with the band at 582 cm^−1^. The strong band at 1344 cm^−1^ indicates a conductive nature of the poly(PADPA) product. Strengthening of the band at 1344 cm^−1^ indicates an increase in the relative amount of delocalized polaron structures and consequently an increase in electrical conductivity of the oligo- or poly(PADPA) products over time. On the other hand, the relative intensity of the bands atypical for standard PANI, at 1633 cm^−1^ and 1420 cm^−1^, decreased with reaction time in relation to the intensity of the mentioned PANI-ES bands. Additional interesting changes occur in the spectrum at *t* = 24 h related to the spectrum at *t* = 1 h: the bands attributed to “atypical segments” at *ν̃* ≈ 1456 cm^−1^ and 1420 cm^−1^ become at *t* = 24 h noticeably weaker compared to the bands of standard PANI-ES (*e.g.*, at *ν̃* = 1603 cm^−1^ and 1344 cm^−1^), the band at *ν̃* = 1633 cm^−1^ disappears, and we observe a new band at *ν̃* = 1652 cm^−1^ (which is also noticeably weaker compared to the bands of ordinary PANI at *ν̃* = 1603 cm^−1^ and 1344 cm^−1^) and a shoulder at *ν̃* = 1578 cm^−1^. The band at *ν̃* = 1652 cm^−1^ could be attributed to the stretching of quinonoid C=O groups/C–C ring-stretching vibrations in phenoxazine-type units^64^ with possible contributions of phenazine- and *N*-phenylphenazine-type units; C=O groups can be formed by the partial hydrolysis of iminoquinonoid C=N bonds.^65^ The shoulder at *ν̃* = 1578 cm^−1^ can also be attributed to substituted phenoxazine- and phenazine-type units.^53^ The bands at *ν̃* = 1652, 1578, and 585 cm^−1^ indicate certain overoxidation of oligo- or poly(PADPA) produced at long reaction times (*t* = 24 h). The relative intensities of the bands attributed to DMSO (at *ν̃* = 1046, 720, and 685 cm^−1^) gradually decrease with reaction time, *e.g.*, the band at *ν̃* = 685 cm^−1^ is very strong and the most intense band in the spectrum at *t* = 5 min, while it is weak at *t* = 24 h. This occurs due to the increase of the amount of oligo- or poly(PADPA) chains (as the reaction proceeds) on the surface of the reaction mixture “drop” which consequently leads to a reduction of surface concentration of DMSO. Thereby, as the reaction proceeds, oligo- or poly(PADPA) chains are becoming dominating species for Raman scattering instead of DMSO. We have observed an increase in the relative intensity of the band at *ν̃* = 603 cm^−1^ (assigned to *δ*(SO_2_)/B ring deformation in PANI-like segments) with an increase in reaction time (it is weak at *t* = 5 min, medium at *t* = 1 h, and strong at *t* = 24 h). This feature could be explained by an increase in oligo- or poly(PADPA) yield, and therefore, an increase in the surface concentration of DBS anions, which could serve as counterions (dopant ions), compensating positive charges of the poly(PADPA) backbone. The prominent increase in the relative intensity of the band at *ν̃* = 582 cm^−1^ with increasing reaction time is observed. At *t* = 24 h, it becomes very strong, bearing an intensity comparable to that of the band at *ν̃* = 1344 cm^−1^. Such band development might come from an increase in the amount of phenoxazine-type units at longer reaction time due to the side processes connected with *ortho*-coupling of PADPA molecules. An additional factor could be an increase in DBS anions surface concentration. It should be noted that bands at similar positions, *ν̃* = 576 cm^−1^ (strong) and *ν̃* ≈ 607 cm^−1^ (weak), are present in the solid-state Raman spectrum of PANI doped with dodecyl benzenesulfonic acid (PANI-DBSA).^68^

**Fig. 9:**
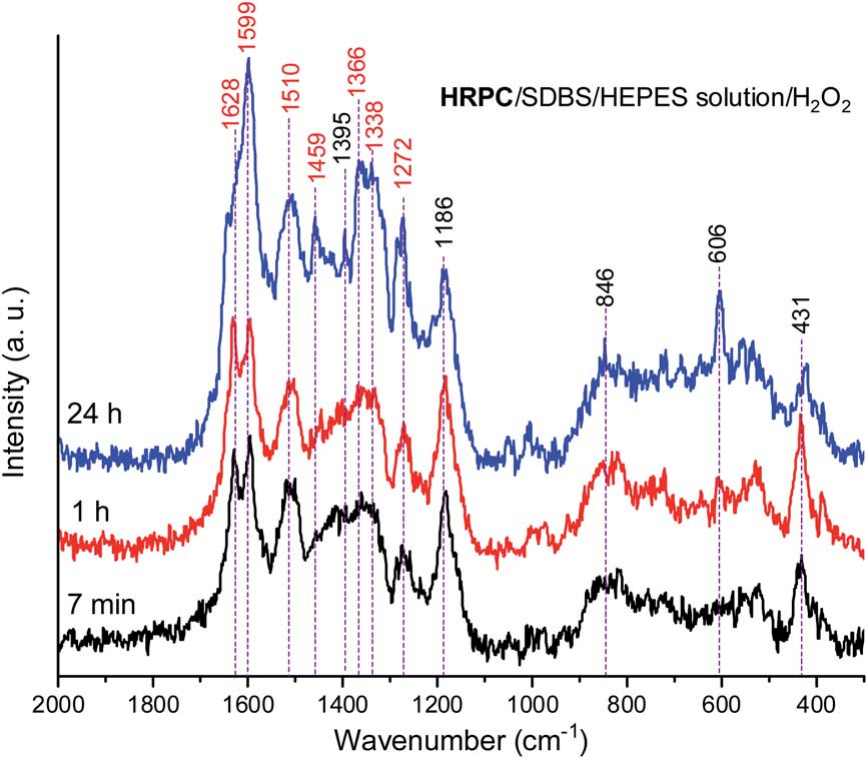
Raman spectra of a reaction mixture prepared with an aqueous 0.1 M HEPES solution at pH 4.3, with [HRPC] = 30.0 nM, [SDBS] = 3.0 mM, [PADPA]_0_ = 1.0 mM, [H_2_O_2_]_0_ = 1.0 mM, recorded at *t* = 7 min, 1 h, and 24 h at RT. The bands characteristic for PANI-ES products are labeled in red, see text; a.u., arbitrary units.

The Raman spectra of the reaction mixture containing HRPC as a catalyst for the reaction times *t* = 7 min, 1 h and 24 h (Fig. 9) qualitatively resemble the Raman spectra obtained with hemin as a catalyst. Nevertheless, there are certain differences which will be specified and discussed in the following.

In both systems, the one containing hemin and the other containing HRPC after a reaction time *t* = 24 h, the bands attributed to standard PANI-ES (*ν̃* ≈ 1600 cm^−1^ and ≈ 1340 cm^−1^) are stronger compared to the bands of atypical units (*ν̃* ≈ 1420–1440 cm^−1^) and both spectra contain a band at *ν̃* = 606 cm^−1^. It is interesting to note that the Raman spectra obtained in the reaction mixture containing HRPC as catalyst do not contain a band at *ν̃* ≈ 580 cm^−1^ (attributable to phenoxazine-type units), and they do not show a strong “phenazine band” at *ν̃* ≈ 1420 cm^−1^ in the initial phase of the reaction (*t* = 7 min), as it is the case for the reaction mixture with hemin as a catalyst. In addition, the spectra of the system with HRPC do not show a band at *ν̃* ≈ 680 cm^−1^, as was the case when hemin was used (Fig. 8). This indicates that the consumption of PADPA and the formation of poly(PADPA) oligo- or polymeric chains at the surface of the reaction mixture “drop” analyzed was faster in the system with HRPC than in the one containing hemin as a catalyst, which was also expected. This is in good agreement with the results from the UV/vis/NIR and EPR measurements (see above). When comparing the Raman spectra recorded at *t* = 24 h of the reaction mixtures with either HRPC or hemin as a catalyst, one more difference is evident. The spectrum for the system with HRPC contains a broad band in the region of the *ν*(C–N^+^) vibrations, with two peaks of similar intensity at around *ν̃* = 1340 cm^−1^ (*ν*(C–N˙^+^)_SQ_ vibration of more delocalized polaron structures) and *ν̃* = 1366 cm^−1^ (*ν*(C–N^+^) ring stretching vibration in substituted *N*-phenylphenazine- and phenoxazine-type units and *ν*(C–N˙^+^)_SQ_ vibration in polaron structures of low delocalization) (Fig. 9), while the spectrum of the system containing hemin (Fig. 8) has a strong *ν*(C–N˙^+^)_SQ_ band, at *ν̃* = 1340 cm^−1^ and a shoulder at ≈ 1370 cm^−1^.

**Fig. 10:**
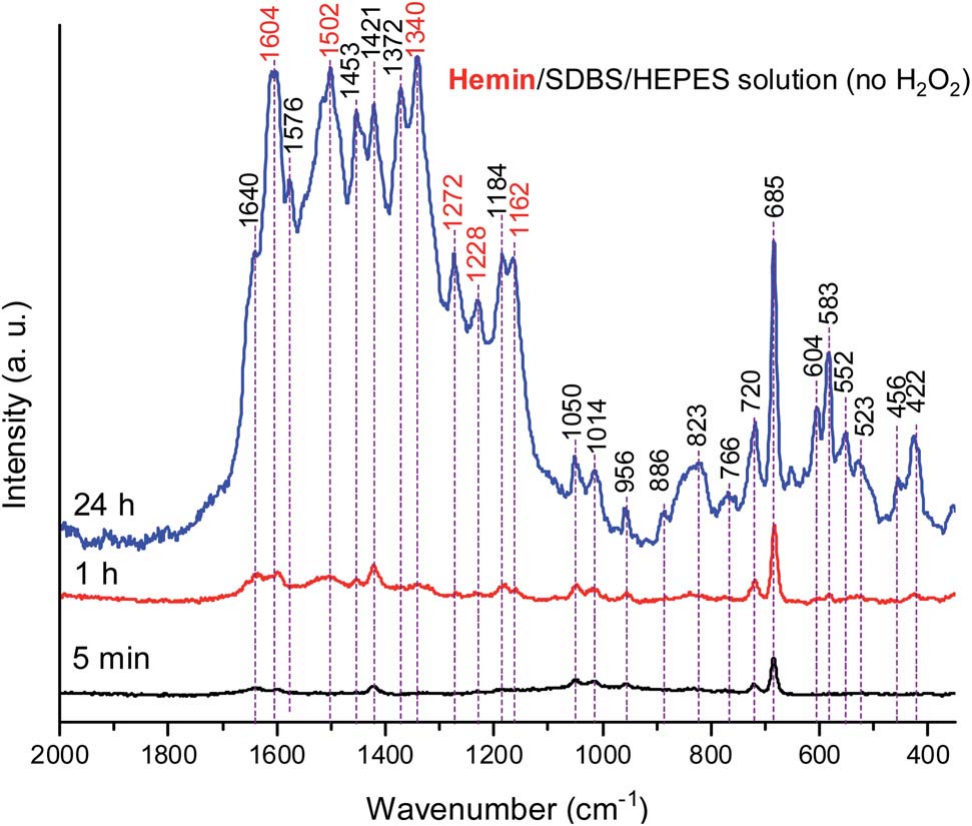
Raman spectra of a reaction mixture prepared with an aqueous 0.1 M HEPES solution at pH 4.3, with [hemin] = 10.0 μM, [SDBS] = 5 mM, [PADPA]_0_ = 1.0 mM, no H_2_O_2_, recorded at *t* = 5 min, 1 h, and 24 h at RT. The bands characteristic for PANI-ES products are labeled in red, see text; a.u., arbitrary units.

Due to the results obtained for the hemin-catalyzed reaction analyzed by UV/vis/NIR and EPR measurements (see above), Raman spectra of the reaction mixture containing hemin without added H_2_O_2_ were also recorded, at *t* = 5 min, 1 h, and 24 h, see Fig. 10.

A comparison of the Raman spectra of the reaction mixtures without H_2_O_2_ (Fig. 10) and with H_2_O_2_ (Fig. 8) – both recorded after *t* = 24 h – shows that the absorption band pattern in both cases is very similar. However, differences in the band intensities are obvious when spectra recorded after the same reaction time are compared. This is directly related to the lower PADPA monomer consumption for the reaction without H_2_O_2_ (lower band intensities) as compared to the one with H_2_O_2_ (higher band intensities).

Comparing the Raman spectra recorded at *t* = 24 h for the two hemin systems (with and without H_2_O_2_) in terms of ratios of intensities of the polaron *ν*(C–N˙^+^)_SQ_ band at *ν̃* ≈ 1340 cm^−1^, which is characteristic of standard conductive PANI-ES, and the bands at *ν̃* ≈ 1453 and 1420 cm^−1^, attributed to segments atypical for standard PANI-ES, *I*_1340_/*I*_1453_, and *I*_1340_/*I*_1420_, respectively, it is clear that these ratios are higher for the hemin system containing H_2_O_2_ than the one without H_2_O_2_. Also, at *t* = 24 h, the bands at *ν̃* = 1372 cm^−1^ and 1576 cm^−1^ are present as distinct bands in the spectrum of the hemin reaction mixture without H_2_O_2_, while in the spectrum of the hemin reaction mixture with H_2_O_2_ they appear as shoulders, only. The bands at *ν̃* = 604 and 583 cm^−1^ have lower relative intensities in the spectrum of the hemin system without H_2_O_2_ compared to these bands in the spectrum of the hemin system with H_2_O_2_.

Although it was not the main focus of the work, Raman spectroscopy measurements were also carried out for reaction mixtures prepared with 0.1 M dihydrogenphosphate solution instead of 0.1 M HEPES solution (both at pH = 4.3), see Fig. S-21–S-24.† As a general observation, the data obtained is in good agreement with the UV/vis/NIR and EPR data for the same reaction conditions and the explanations given above.

### 3.3. Discussion of the main points of interest

#### 3.3.1. The use of hemin instead of HRPC

Our investigations have shown that HRPC can be replaced by hemin for the synthesis of PANI-ES-like products using PADPA as monomer at an initial concentration of 1.0 mM (and 1.0 mM H_2_O_2_) in an aqueous solution containing SDBS micelles (5.0 mM SDBS) at pH = 4.3 (0.1 M HEPES). High conversions of PADPA were achieved within a reaction time of *t* = 24 h at RT. All three complementary spectroscopic measurements used (UV/vis/NIR, EPR, and Raman spectroscopy), indicate that the products contain stable radicals that are typical for the conductive polaron state of PANI-ES (Fig. 1). However, due to the significantly lower catalytic activity of hemin as compared to HRPC, the molar amount of catalyst used in our experiments (10 μM hemin) was significantly higher as compared to the concentration of HRPC (30 nM). Nevertheless, the costs for hemin to yield comparable PADPA conversions within a similar reaction time as with HRPC were only about 5% of the costs for HRPC, see ESI,† Section 6. An interesting topic of a possible follow-up investigation would be to explore whether hemin's catalytic activity could be increased by addition of electron-donating groups (*e.g.*, histidine or imidazole) at a concentration that optimizes peroxide coordination to Fe(iii) of hemin. Although the basic form of hemin, hematin (= porFe^III^-OH), was used before as catalyst in aqueous micellar solutions of SDBS at *T* = 4 °C for the synthesis of polypyrrole,^69^ our work is the first one on the formation of poly(PADPA) products that resemble PANI-ES by using hemin as catalyst.

#### 3.3.2. The role of SDBS micelles

There are two roles of SDBS micelles in the hemin-catalyzed formation of PANI-ES like products from PADPA: (i) the micelles prevent hemin aggregation, which enables hemin to be catalytically active, and (ii) the micelles serve as reaction “template”, *i.e.*, they guide the reaction towards the desired formation of the emeraldine salt form of PANI. Since for this latter role, which is directly related to the chosen reaction, the negative surface charge of the micelles is essential.^9^ Whether other types of micelles (cationic, zwitterionic, or neutral) could be used as well for hemin-catalyzed reactions that do not rely on a “template” effect, remains to be investigated. In other words, one key question of interest is, whether there is a correlation between micelle type, capability to prevent hemin aggregation, and catalytic activity of hemin for (simple) reactions involving substrates that are different from PADPA and do not involve complex follow-up reactions that occur uncatalyzed after substrate oxidation as in the case of PADPA.

#### 3.3.3. The use of HEPES *vs.* dihydrogenphosphate

In the hemin-catalyzed reactions investigated in this work, the use of HEPES proved to be more advantageous than the use of dihydrogenphosphate. Considering that the spectra of hemin in these two salt solutions look very similar in the presence of SDBS micelles (Fig. S-3†), one can conclude that in both systems the fraction of monomeric hemin is very similar. Therefore, the difference in the catalytic activity of hemin in the two micellar solutions is not due to different physical states of hemin (monomer, π–π dimer, *etc.*). The difference more likely originates from different iron coordination propensities of the two salt species. It has already been shown that dihydrogenphosphate can displace water and bind to Fe(iii) of hemin.^70^ From the results we obtained, it appears that the binding of H_2_PO_4_^−^ is so strong that hydrogen peroxide is unable to displace the coordinated dihydrogenphosphate (or displaces it to a very low extent only), making the oxidation of hemin with H_2_O_2_ to form compound I inefficient (Fig. S-10 and S-13†). On the other hand, when HEPES was used, the impact of hydrogen peroxide was significant (Fig. 2 and S-12†). Therefore, we conclude that under the conditions used HEPES does not block the 5^th^ and 6^th^ coordination sites of Fe(iii) as strong as dihydrogenphosphate does. In addition, HEPES species that are partially coordinated to Fe(iii) may promote the reaction by either weak electron donation to the iron of hemin or by stabilizing a transition state.

#### 3.3.4. The role of H_2_O_2_

As already stated in the Introduction, the aim of our work was to try to replace HRPC by its prosthetic group (ferric heme *b* = hemin). More specifically, we asked ourselves whether hemin can be used as peroxidase-mimic, as catalyst system in which hemin in SDBS micelles in the presence of H_2_O_2_ follows the catalytic heme peroxidase cycle (Scheme S-1†), but in a less efficient manner. Although hemin proved to catalyze the formation of PANI-ES like material, it is by no means clear whether the observed reactions with hemin in the micellar system followed the same peroxidase cycle, with intermediate formation of compound I and compound II. Exploring the mechanism of the hemin/H_2_O_2_-catalyzed PADPA oxidation in the presence of SDBS micelles may be a challenge that could be addressed in a future study.

So far, important control measurements of reactions run without H_2_O_2_ clearly demonstrated that the hemin-catalyzed formation of PANI-ES-like products from PADPA in 0.1 M HEPES solution at pH = 4.3 (in the presence of SDBS micelles) also occurs to some extent without H_2_O_2_ (Fig. 2), although the reaction is much slower than with H_2_O_2_ (Fig. 5 and S-12†). It seems that the oxidation of PADPA (or TMB) with hemin as catalyst occurs *via* two mechanisms, one involving H_2_O_2_ as terminal oxidant, the other with O_2_ (which was present in all reaction mixtures due to their exposure to air). Preliminary measurements with PADPA as substrate were carried out without H_2_O_2_ under “oxygen-free” conditions (see Fig. S-25, S-26 and accompanying text for details†). The observation made is that in the absence of H_2_O_2_ oxygen is necessary for the reaction to proceed. Although the mechanism involving oxygen is not clear at the moment and deserves further investigations, one possibility how the reaction might proceed is that Fe(iii)PPIX is reduced to Fe(ii)PPIX by PADPA, while at the same time PADPA is oxidized to the PADPA radical. Fe(ii)PPIX is then reoxidized to Fe(iii)PPIX by oxygen.||||Heme Fe^2+^ undergoes autooxidation in the presence of oxygen to Fe^3+^.^79^ Alternatively, the mechanism might be completely different, and it may include the formation of hydrogen peroxide *in situ*.^71^

## 4. Conclusion and outlook

The work carried out showed that hemin can be used successfully as catalyst for the oxidation of PADPA in a slightly acidic aqueous micellar solution of SDBS to yield products with PANI-ES structural units that are typical for the conductive form of polyaniline. Apart from applications of the products obtained, future studies may involve the investigation of the binding of hemin to the micelles, the use of other surfactant aggregates, or an investigation of the reaction mechanism and a possible substrate selectivity. Beyond this, experimental proof of catalytically active hemin in aggregates of amphiphiles is also of interest for origin-of-life research. Porphyrins like PPIX are molecules that are not only ubiquitously present as prosthetic groups in contemporary proteins,^72^ but may have been present already in prebiotic times.^73,74^ Due to the likely prebiotic existence of simple membrane-forming amphiphiles,^75^ these amphiphiles might have associated with metal porphyrins to form catalytically active assemblies for accelerating localized chemical transformations. In this way, amphiphile-heme aggregates could have served as a kind of “primitive enzymes”, even before the suggested heme RNA quadruplex systems possibly formed.^76^ Although this is pure speculation, the idea is worth considering in scenarios on the prebiological emergence of functional compartment systems, in steps that preceded the first living cells at the origin of life on earth.^77^

## Conflicts of interest

There is no conflict of interest to declare.

## Supplementary Material

RA-012-D2RA02198F-s001

## References

[cit1] Junker K., Zandomeneghi G., Guo Z., Kissner R., Ishikawa T., Kohlbrecher J., Walde P. (2012). RSC Adv..

[cit2] Wang R., Huang X. (2021). ACS Omega.

[cit3] Rumbau V., Pomposo J. A., Alduncin J. A., Grande H., Mecerreyes D., Ochoteco E. (2007). Enzyme Microb. Technol..

[cit4] Liu W., Cholli A. L., Nagarajan R., Kumar J., Tripathy S., Bruno F. F., Samuelson L. (1999). J. Am. Chem. Soc..

[cit5] Luginbühl S., Milojević-Rakić M., Junker K., Bajuk-Bogdanović D., Pašti I., Kissner R., Ćirić-Marjanović G., Walde P. (2017). Synth. Met..

[cit6] Zhang Y., Serrano-Luginbühl S., Kissner R., Milojević-Rakić M., Bajuk-Bogdanović D., Ćirić-Marjanović G., Wang Q., Walde P. (2018). Langmuir.

[cit7] Liu W., Kumar J., Tripathy S., Samuelson L. A. (2002). Langmuir.

[cit8] Liu W., Kumar J., Tripathy S., Senecal K. J., Samuelson L. (1999). J. Am. Chem. Soc..

[cit9] Walde P., Guo Z. (2011). Soft Matter.

[cit10] Otrokhov G. V., Morozova O. V., Vasil’eva I. S., Shumakovich G. P., Zaitseva E. A., Khlupova M. E., Yaropolov A. I. (2013). Biochemistry (Moscow).

[cit11] Janoševic Ležaić A., Luginbühl S., Bajuk-Bogdanović D., Pašti I., Kissner R., Rakvin B., Walde P., Ćirić-Marjanović G. (2016). Sci. Rep..

[cit12] Xu P. , Singh A. and Kaplan D. L., Enzymatic catalysis in the synthesis of polyanilines and derivatives of polyanilines, Springer, Berlin, Heidelberg, 2006

[cit13] Kurisu M., Aoki H., Jimbo T., Sakuma Y., Imai M., Serrano-Luginbühl S., Walde P. (2019). Commun. Chem..

[cit14] Fujisaki T., Kashima K., Serrano-Luginbühl S., Kissner R., Bajuk-Bogdanović D., Milojević-Rakić M., Ćirić-Marjanović G., Busato S., Lizundia E., Walde P. (2019). RSC Adv..

[cit15] Guo Z., Rüegger H., Kissner R., Ishikawa T., Willeke M., Walde P. (2009). Langmuir.

[cit16] Dunford H. B., Stillman J. S. (1976). Coord. Chem. Rev..

[cit17] Asher C., de Villiers K. A., Egan T. J. (2009). Inorg. Chem..

[cit18] Kuter D., Venter G. A., Naidoo K. J., Egan T. J. (2012). Inorg. Chem..

[cit19] Kuter D., Streltsov V., Davydova N., Venter G. A., Naidoo K. J., Egan T. J. (2014). Inorg. Chem..

[cit20] Berglund G. I., Carlsson G. H., Smith A. T., Szöke H., Henriksen A., Hajdu J. (2002). Nature.

[cit21] Dunford H. B. , Peroxidases and catalases: biochemistry, biophysics, biotechnology and physiology, John Wiley & Sons, 2010

[cit22] Poulos T. L. (2014). Chem. Rev..

[cit23] Huang X., Groves J. T. (2018). Chem. Rev..

[cit24] Bertini G. , Gray H. B., Gray H., Valentine J. S., Stiefel E. I. and Stiefel E., Biological inorganic chemistry: structure and reactivity, University Science Books, 2007

[cit25] Mazumdar S. (1990). J. Phys. Chem..

[cit26] Mazumdar S. and Mitra S., in Structures and Biological Effects (Structure and Bonding, vol. 81), Springer-Verlag, Berlin, Heidelberg, 1993, pp. 115–145

[cit27] Das D. K., Medhi O. K. (2005). Indian J. Chem..

[cit28] Simplicio J. (1972). Biochemistry.

[cit29] Mazumdar S., Medhi O. K., Mitra S. (1988). Inorg. Chem..

[cit30] Boffi A., Das T. K., della Longa S., Spagnuolo C., Rousseau D. L. (1999). Biophys. J..

[cit31] Beaven G. H., Chen S.-H., D'Albis A., Gratzer W. B. (1974). Eur. J. Biochem..

[cit32] Monzani E., Bonafè B., Fallarini A., Redaelli C., Casella L., Minchiotti L., Galliano M. (2001). Biochim. Biophys. Acta, Protein Struct. Mol. Enzymol..

[cit33] Ascenzi P., di Masi A., Fanali G., Fasano M. (2015). Cell Death Discovery.

[cit34] Travascio P., Bennet A. J., Wang D. Y., Sen D. (1999). Chem. Biol..

[cit35] Travascio P., Witting P. K., Mauk A. G., Sen D. (2001). J. Am. Chem. Soc..

[cit36] Sen D., Poon L. C. H. (2011). Crit. Rev. Biochem. Mol. Biol..

[cit37] Stefan L., Denat F., Monchaud D. (2012). Nucleic Acids Res..

[cit38] Hagiwara S., Momotake A., Ogura T., Yanagisawa S., Suzuki A., Neya S., Yamamoto Y. (2021). Inorg. Chem..

[cit39] Shen W., Deng H., Gao Z. (2014). RSC Adv..

[cit40] Derat E., Shaik S. (2006). J. Am. Chem. Soc..

[cit41] Derat E., Shaik S. (2006). J. Am. Chem. Soc..

[cit42] Derat E., Shaik S., Rovira C., Vidossich P., Alfonso-Prieto M. (2007). J. Am. Chem. Soc..

[cit43] Campomanes P., Rothlisberger U., Alfonso-Prieto M., Rovira C. (2015). J. Am. Chem. Soc..

[cit44] Veitch N. C., Smith A. T. (2000). Adv. Inorg. Chem..

[cit45] Penner-Hahn J. E., Smith Eble K., McMurry T. J., Renner M., Balch A. L., Groves J. T., Dawson J. H., Hodgson K. O. (1986). J. Am. Chem. Soc..

[cit46] Ator M. A., David S. K., Ortiz de Montellano P. R. (1987). J. Biol. Chem..

[cit47] Gumiero A., Murphy E. J., Metcalfe C. L., Moody P. C., Raven E. L. (2010). Arch. Biochem. Biophys..

[cit48] Ortmayer M., Fisher K., Basran J., Wolde-Michael E. M., Heyes D. J., Levy C., Lovelock S. L., Anderson J. R., Raven E. L., Hay S. (2020). ACS Catal..

[cit49] do Nascimento G. M. and Souza M. A. d., Spectroscopy of nanostructured conductive polymers, in Nanostructured Conductive Polymers, John Wiley and Sons, Chichester, 2015

[cit50] Kulikov A. V., Bogatyrenko V. R., Belonogova O. V., Fokeeva L. S., Lebedev A. V., Echmaeva T. A., Shunina I. G. (2002). Russ. Chem. Bull..

[cit51] Krinichnyi V. I., Roth H. K., Schrödner M., Wessling B. (2006). Polymer.

[cit52] Junker K., Luginbühl S., Schüttel M., Bertschi L., Kissner R., Schuler L. D., Rakvin B., Walde P. (2014). ACS Catal..

[cit53] Ćirić-Marjanović G., Trchová M., Stejskal J. (2008). J. Raman Spectrosc..

[cit54] Travascio P., Li Y., Sen D. (1998). Chem. Biol..

[cit55] Śledź R. K. P., Chruszcz M., Zimmerman M. D., Minor W., Woźniak K. (2010). Acta Crystallogr., Sect. B: Struct. Sci..

[cit56] de Villiers K. A., Kaschula C. H., Egan T. J., Marques H. M. (2007). JBIC, J. Biol. Inorg. Chem..

[cit57] Moosavi-Movahedi A. A., Semsarha F., Heli H., Nazari K., Ghourchian H., Hong J., Hakimelahi G. H., Saboury A. A., Sefidbakht Y. (2008). Colloids Surf., A.

[cit58] Moosavi-Movahedi Z., Gharibi H., Hadi-Alijanvand H., Akbarzadeh M., Esmaili M., Atri M. S., Sefidbakht Y., Bohlooli M., Nazari K., Javadian S., Hong J., Saboury A. A., Sheibani N., Moosavi-Movahedi A. A. (2015). J. Biomol. Struct. Dyn..

[cit59] Moosavi-Movahedi Z., Kalejahi E. S., Nourisefat M., Maghami P., Poursasan N., Moosavi-Movahedi A. A. (2017). Colloids Surf., A.

[cit60] Walde P., Kashima K., Ćirić-Marjanović G. (2019). Front. Bioeng. Biotechnol..

[cit61] Dmitrieva E., Dunsch L. (2011). J. Phys. Chem. B.

[cit62] Dennany L., Innis P. C., McGovern S. T., Wallace G. G., Forster R. J. (2011). Phys.
Chem. Chem. Phys..

[cit63] do Nascimento G. M., Pereira da Silva J. E., Córdoba de Torresi S. I., Temperini M. L. A. (2002). Macromolecules.

[cit64] Brolo A. G., Sanderson A. C. (2004). Can. J. Chem..

[cit65] Ćirić-Marjanović G., Trchová M., Konyushenko E. N., Holler P., Stejskal J. (2008). J. Phys. Chem. B.

[cit66] Socrates G. , Infrared and Raman characteristic group frequencies: tables and charts, John Wiley & Sons, 2004

[cit67] Cochet M., Louarn G., Quillard S., Boyer M. I., Buisson J. P., Lefrant S. (2000). J. Raman Spectrosc..

[cit68] Shakoor A., Rizvi T. Z. (2010). J. Raman Spectrosc..

[cit69] Ravichandran S., Nagarajan S., Kokil A., Ponrathnam T., Bouldin R. M., Bruno F. F., Samuelson L., Kumar J., Nagarajan R. (2012). Langmuir.

[cit70] Márquez I., Olloqui-Sariego J. L., Molero M., Andreu R., Roldán E., Calvente J. J. (2021). Inorg. Chem..

[cit71] Brezny A. C., Nedzbala H. S., Mayer J. M. (2021). Chem. Commun..

[cit72] Sitte E., Senge M. O. (2020). Eur. J. Org Chem..

[cit73] Pleyer H. L., Strasdeit H., Fox S. (2018). Origins Life Evol. Biospheres.

[cit74] Fox S., Strasdeit H. (2013). Astrobiology.

[cit75] Deamer D., Dworkin J. P., Sandford S. A., Bernstein M. P., Allamandola L. J. (2002). Astrobiology.

[cit76] Anastasi C., Buchet F. F., Crowe M. A., Parkes A. L., Powner M. W., Smith J. M., Sutherland J. D. (2007). Chem. Biodiversity.

[cit77] Monnard P.-A., Walde P. (2015). Life.

[cit78] Puustinen A., Wikström M. (1991). Proc. Natl. Acad. Sci. U. S. A..

[cit79] Mollan T. L., Alayash A. I. (2013). Antioxid. Redox Signaling.

