# Supplementary material for: Hemin-catalyzed oxidative oligomerization of p-aminodiphenylamine (PADPA) in the presence of aqueous sodium dodecylbenzenesulfonate (SDBS) micelles
Source: RSC Adv. 2022 May 3;12(21):13154–67. doi: 10.1039/d2ra02198f (PMC9063397; doi:10.1039/d2ra02198f)
Supplement: RA-012-D2RA02198F-s001 [file RA-012-D2RA02198F-s001.pdf]

Electronic Supplementary Material (ESI) for RSC Advances.
This journal is © The Royal Society of Chemistry 2022

Electronic Supporting Information (ESI)

Hemin-catalyzed oxidative oligomerization of *p*-aminodiphenylamine (PADPA) in the presence of aqueous sodium dodecylbenzenesulfonate (SDBS) micelles

Nemanja Cvjetan,[a] Reinhard Kissner,[b] Danica Bajuk-Bogdanović,[c] Gordana Ćirić-Marjanović[c] and Peter Walde[a,*]

[a] Department of Materials, Laboratory for Multifunctional Materials, ETH Zürich, Vladimir-Prelog-Weg 5, 8093 Zürich, Switzerland. E-mail: peter.walde@mat.ethz.ch

[b] Department of Chemistry and Applied Biosciences, Laboratory of Inorganic Chemistry, Vladimir-Prelog-Weg 2, 8093 Zürich, Switzerland

[c] Faculty of Physical Chemistry, University of Belgrade, Studentski trg 12-16, 11158 Belgrade, Serbia

*Corresponding Author:
E-mail: peter.walde@mat.ethz.ch

CONTENT

**1. Peroxidase cycle of heme peroxidases like HRPC**		
Schematic representation of the three steps of the peroxidase cycle of HRPC	**Scheme S-1**	p. S-4
**2. UV/vis/NIR measurements**		
Effect of SDBS on the hemin/H_2O_2-catalyzed oxidation of PADPA in HEPES solution	**Fig. S-1**	p. S-5
Effect of SDBS on the HRPC/H_2O_2-catalyzed oxidation of PADPA in HEPES solution	**Fig. S-2**	p. S-6
UV/vis absorption spectrum of hemin at pH = 4.3 in the presence of SDBS micelles in either HEPES or dihydrogenphosphate solution	**Fig. S-3**	p. S-7
Comparison of the UV/vis/NIR absorption spectrum of the oligo- and poly(PADPA) reaction products obtained at pH = 4.3 with HRPC/H_2O_2 in the presence of SDBS micelles with previous data for the same reaction in the presence of AOT vesicles	**Fig. S-4**	p. S-8
Comparison of the UV/vis/NIR absorption spectrum of reaction mixtures containing SDBS micelles, PADPA, H_2O_2, and either hemin or HRPC prepared with either HEPES or dihydrogenphosphate solution at pH = 4.3, reacted for t = 24 h at RT	**Fig. S-5**	p. S-9
Comparison of the activity of hemin in the presence of SDBS micelles, H_2O_2, and either 0.1 M HEPES or 0.1 M dihydrogenphosphate at pH = 4.3 using the TMB assay	**Fig. S-6**	p. S-10
Control measurements for the HRPC/HEPES system	**Fig. S-7**	p. S-11
Control measurements for the hemin/HEPES system	**Fig. S-8**	p. S-12
Comparison of the hemin/HEPES and HRPC/HEPES systems with and without H_2O_2	**Fig. S-9**	p. S-13
Control measurements for the hemin/dihydrogenphosphate system	**Fig. S-10**	p. S-14
Control measurements for the HRPC/dihydrogenphosphate system	**Fig. S-11**	p. S-15
Control measurements for the hemin/HEPES system using the TMB assay	**Fig. S-12**	p. S-16
Control measurements for the hemin/dihydrogenphosphate system using the TMB assay	**Fig. S-13**	p. S-17
Time-dependent changes of the UV/vis/NIR absorption spectrum of the reaction mixture prepared with the HEPES solution at pH = 4.3 and at RT, containing hemin, PADPA, SDBS micelles and H_2O_2 ("optimal reaction conditions" for hemin)	**Fig. S-14**	p. S-18
Time-dependent changes of the UV/vis/NIR absorption spectrum of the reaction mixture prepared with the HEPES solution at pH = 4.3 and at RT, containing HRPC, PADPA, SDBS micelles and H_2O_2 ("optimal reaction conditions" for HRPC)	**Fig. S-15**	p. S-19

Time-dependent changes of the UV/vis/NIR absorption spectrum of the reaction mixture prepared with the HEPES solution at pH = 4.3 and at RT, containing hemin, PADPA, and SDBS micelles without H_2O_2.	**Fig. S-16**	p. S-20
Time-dependent changes of the UV/vis/NIR absorption spectrum of the reaction mixture prepared with the dihydrogenphosphate solution at pH = 4.3 and at RT, containing hemin, PADPA, SDBS micelles and H_2O_2	**Fig. S-17**	p. S-21
Time-dependent changes of the UV/vis/NIR absorption spectrum of the reaction mixture prepared with the dihydrogenphosphate solution at pH = 4.3 and at RT, containing hemin, PADPA, and SDBS micelles without H_2O_2.	**Fig. S-18**	p. S-22
Comparison of the time-dependent changes of A_{1030} upon oxidation of PADPA in different reaction mixtures containing hemin or HRPC as catalyst at pH = 4.3 and RT.	**Fig. S-19**	p. S-23
**3. EPR spectroscopy measurements**		
Analysis of reaction mixtures prepared with 0.1 M dihydrogenphosphate, pH = 4.3	**Fig. S-20**	p. S-24
**4. Raman spectroscopy measurements**		
Raman spectroscopy analysis of the oligo- and poly(PADPA) products obtained with hemin in the presence of SDBS micelles and H_2O_2, prepared with 0.1 M dihydrogenphosphate, pH = 4.3	**Fig. S-21**	p. S-25
Raman spectroscopy analysis of the oligo- and poly(PADPA) products obtained with hemin in the presence of SDBS micelles without H_2O_2, prepared with 0.1 M dihydrogenphosphate, pH = 4.3	**Fig. S-22**	p. S-26
Comparison of the Raman spectra recorded after a reaction time t = 24 h at RT of reaction mixtures containing hemin as catalyst, prepared either in HEPES or in dihydrogenphosphate solution, with or without H_2O_2.	**Fig. S-23**	p. S-27
Raman spectroscopy analysis of the oligo- and poly(PADPA) products obtained with HRPC in the presence of SDBS micelles and H_2O_2, prepared with 0.1 M dihydrogenphosphate, pH = 4.3	**Fig. S-24**	p. S-28
**5. Preliminary investigations of an "O_2-free" reaction mixture**		
UV/vis/NIR absorption spectrum of a reaction mixture that did not contain H_2O_2 and from which O_2 was removed (measured after t = 24 h)	**Fig. S-25**	p. S-29
Time-dependent changes of the UV/vis/NIR spectrum of a reaction mixture that did not contain H_2O_2 and from which O_2 was first removed and then added again	**Fig. S-26**	p. S-30
**6. Estimation of catalyst cost reduction when HRPC is replaced by hemin for the formation of PANI-ES products from PADPA**		
Calculations made on the basis of the amounts of catalyst used and the price paid for the catalysts		p. S-31
**7. References**		p. S-31

1. Peroxidase cycle of heme peroxidases like HRPC

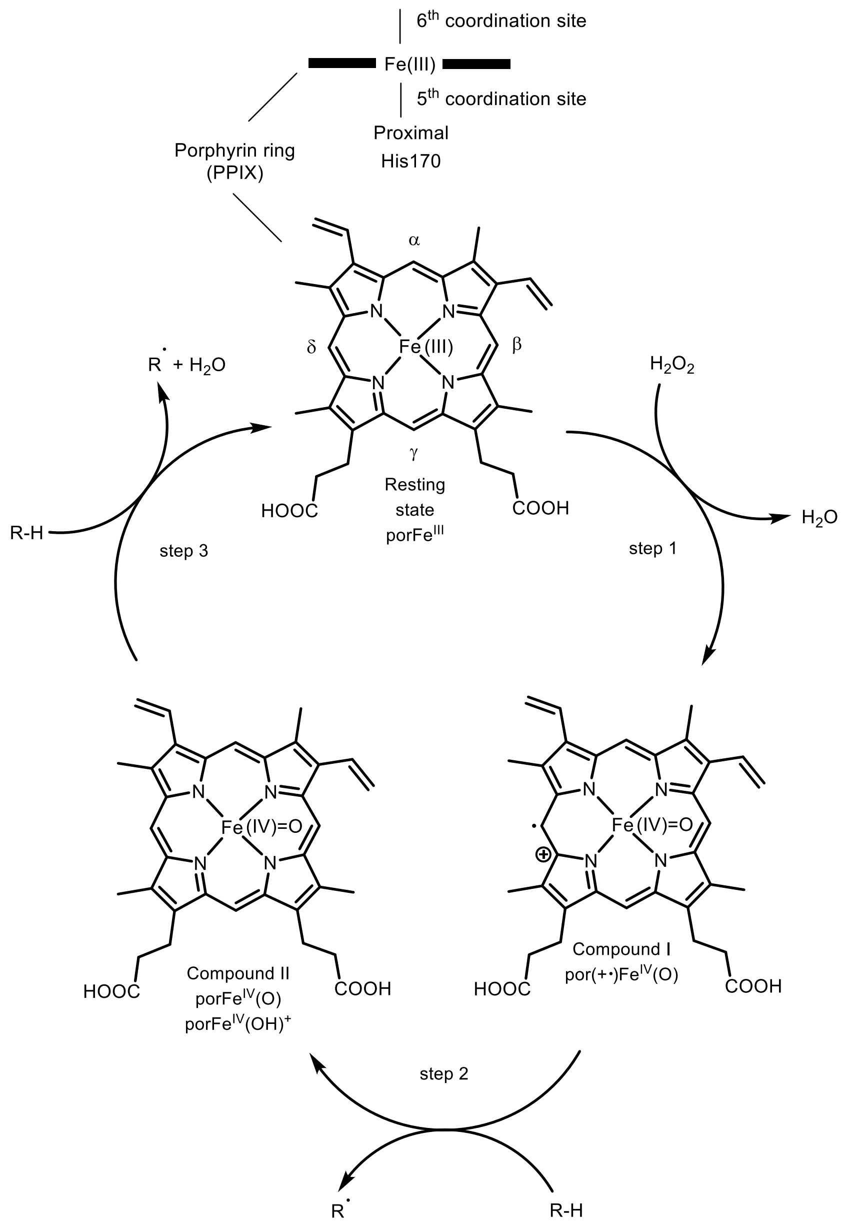

Scheme S-1. Schematic representation of the three steps of the peroxidase cycle of heme peroxidases like HRPC, see for example Junker et al. (2013).[S1] A cross-sectional view of heme *b* in the resting enzyme is shown on top with an indication of the 5th and 6th coordination sites of the iron atom. In HRPC, the 5th coordination site is ligated to His170, the proximal histidine residue, which "pushes" electrons to the iron atom.[S2] Step 1 is the two-electron oxidation of the peroxidase in its resting state (with the ferric heme group abbreviated as porFe^{III}, por = porphyrin) by H_2O_2, which yields Compound I (por(+•)Fe^{IV}(O), with a π-cation radical on the porphyrin ring). In step 2, Compound I oxidizes the reducing substrate (R-H, PADPA in the present case) in the solvent-exposed δ-region of the heme group in a one-electron oxidation reaction to yield the substrate radical R$^{•}$ (a PADPA radical) and Compound II (porFe^{IV}(O) or porFe^{IV}(OH)$^{+}$.[S3,S4] With step 3 - another one-electron oxidation of R-H (PADPA) at the δ-region of the heme group - the cycle is closed and the resting state of HRPC is again obtained.[S2,S5]

2. UV/vis/NIR spectroscopy measurements

Effect of SDBS on the hemin/H_2O_2-catalyzed oxidation of PADPA in HEPES solution

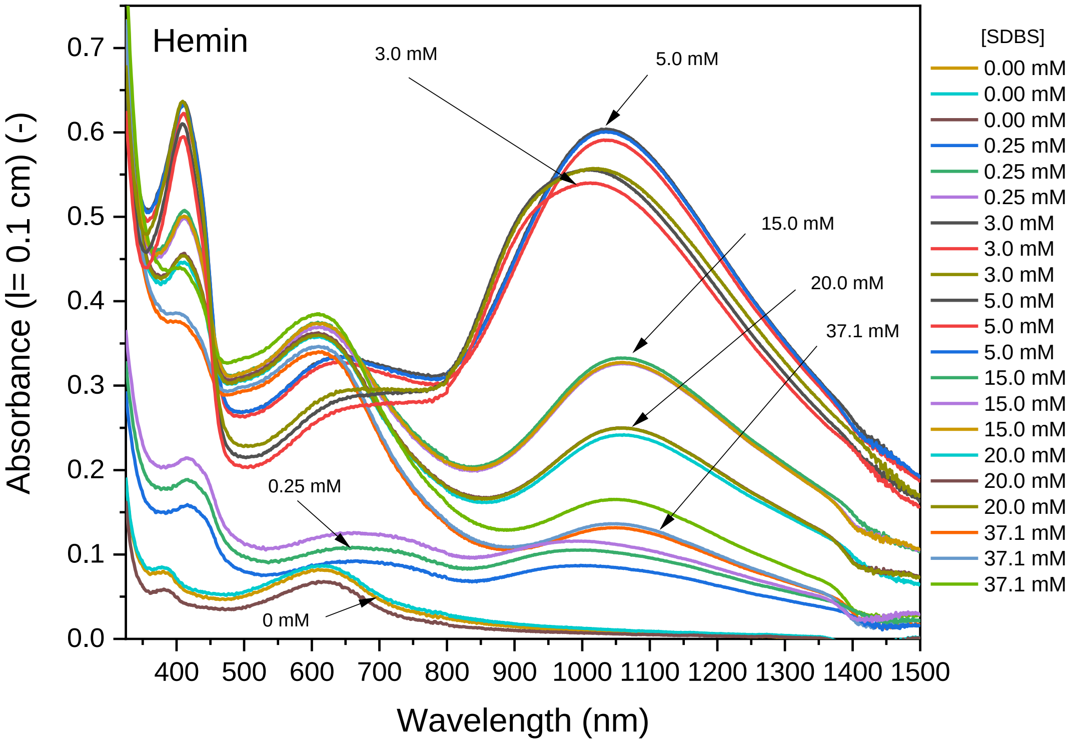

Figure S-1 UV/vis/NIR absorption spectra measured after t = 24 h at RT for reaction mixtures containing hemin as catalyst and different amounts of SDBS.

Reaction conditions:
0.1 M HEPES solution, pH = 4.3; [hemin] = 10 µM, $[PADPA]_0$ = 1.0 mM; $[H_2O_2]_0$ = 1.0 mM. [SDBS] = 0, 0.25, 1.0, 3.0, 5.0, 15.0, 20.0 or 37.1 mM. For each condition, the reactions were run in triplicates.

Notes:
(a) The contribution of hemin to A_{400} (l = 0.1 cm) was ≤ 0.066.
(b) Samples containing 1.0 mM SDBS were turbid even after a reaction time of t = 24 h. Therefore, reliable UV/vis/NIR measurements were not possible.

Effect of SDBS on the HRPC/H_2O_2-catalyzed oxidation of PADPA in HEPES solution

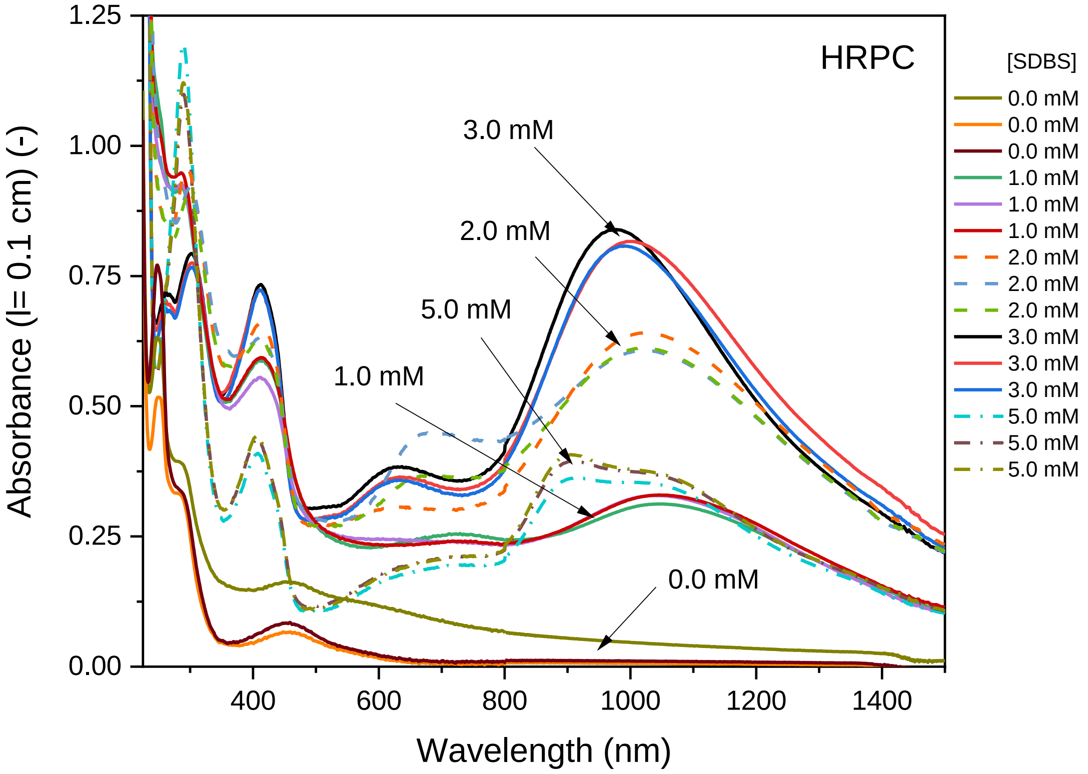

Figure S-2 UV/vis/NIR absorption spectra measured after t = 24 h at RT for reaction mixtures containing HRPC as catalyst and different amounts of SDBS.

Reaction conditions:
0.1 M HEPES solution, pH = 4.3; [HRPC] = 30 nM, $[PADPA]_0$ = 1.0 mM; $[H_2O_2]_0$ = 1.0 mM. [SDBS] = 1.0, 2.0, 3.0 or 5.0 mM. For each condition, the reactions were run in triplicates.

UV/vis absorption spectrum of hemin at pH = 4.3 in the presence of SDBS micelles in either HEPES or dihydrogenphosphate solution

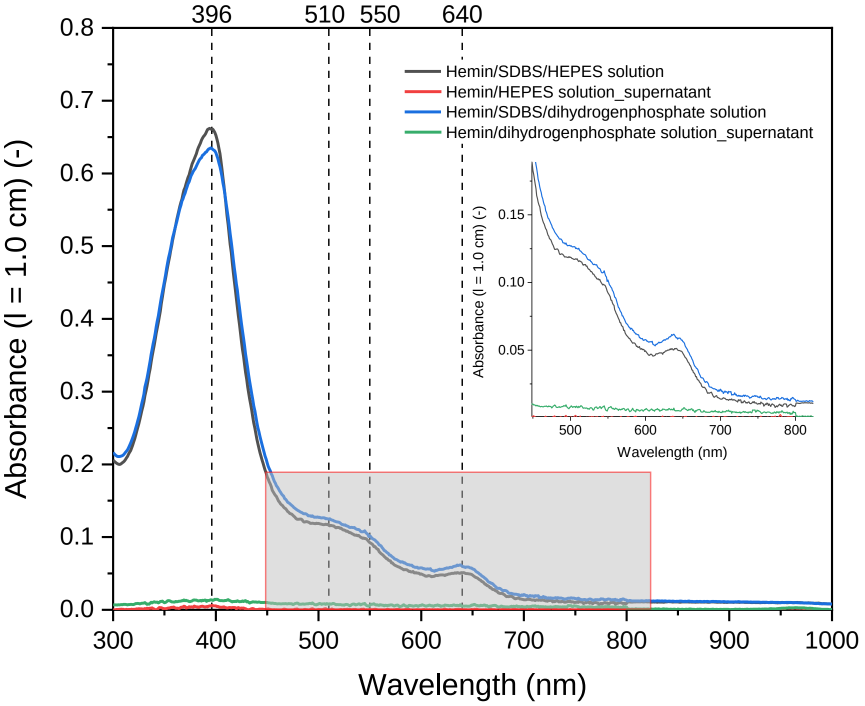

1 2 3 4

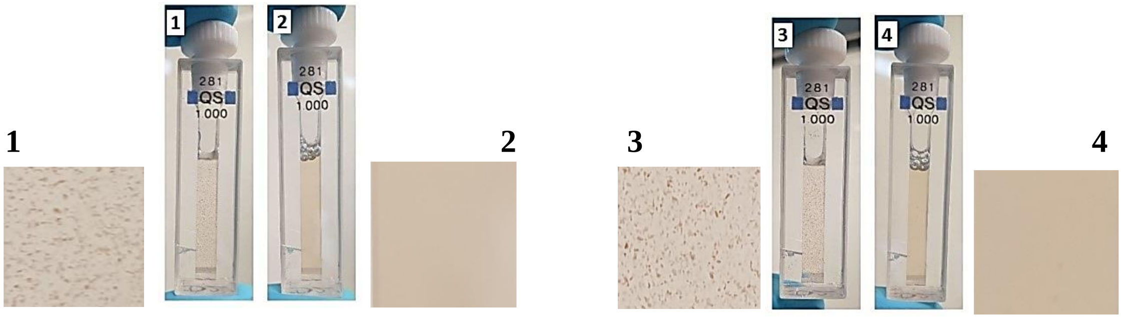

SDBS

without with without with

in HEPES solution in dihydrogenphosphate solution

Figure S-3 *Top:* UV/vis absorption spectrum of 10 µM hemin in the presence of 5.0 mM SDBS in either 0.1 M HEPES solution (black line, A_{396} (1.0 cm) ≈ 0.66; A_{500} (1.0 cm) ≈ 0.12) or 0.1 M dihydrogenphosphate solution (blue line) at pH 4.3. *Inset:* Zoom-in of the region of the Q-bands. *Bottom:* Photographs of the samples prepared, either with the HEPES solution (1,2) without SDBS (1) or with SDBS (2); or prepared with the dihydrogenphosphate solution (3,4) without (3) or with SDBS (4). In the absence of SDBS, hemin aggregates and forms small particles (see zoom-ins). After centrifugation of the SDBS-free solutions (1 and 3), the absorption spectrum of the supernatant was recorded (green and red lines), indicating that hemin precipitated almost quantitatively.

Comparison of the UV/vis/NIR absorption spectrum of the oligo- and poly(PADPA) reaction products obtained at pH = 4.3 with HRPC/H_2O_2 in the presence of SDBS micelles with previous data for the same reaction in the presence of AOT vesicles

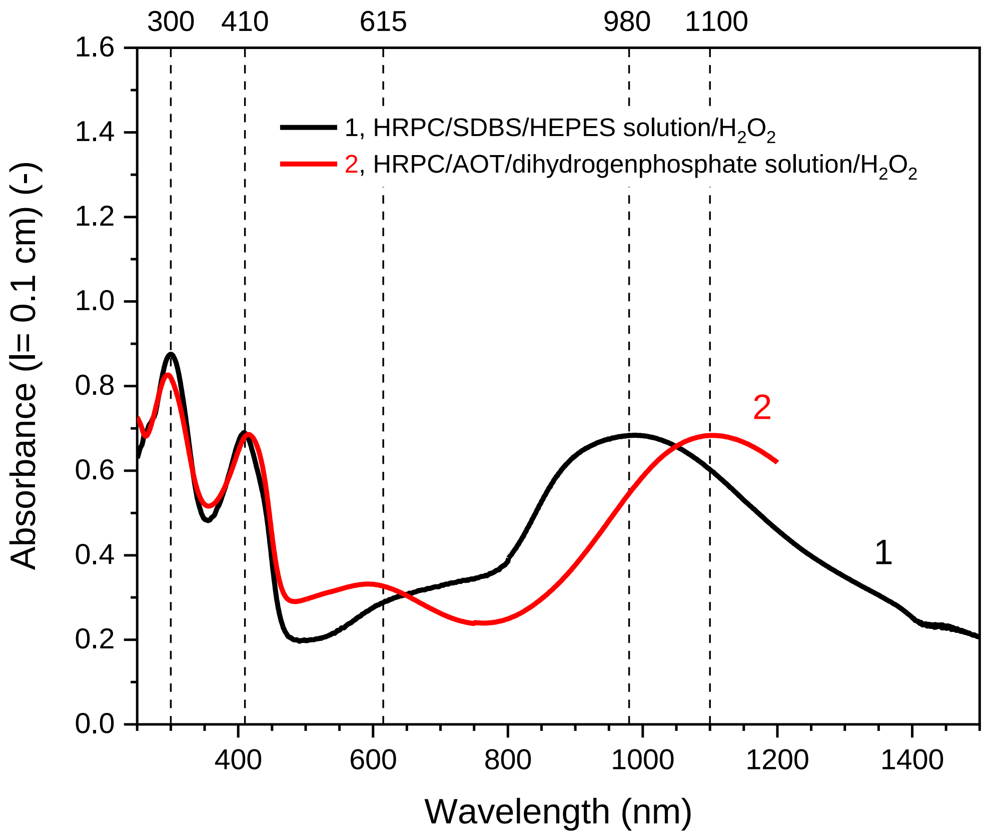

Figure S-4 Comparison of the UV/vis/NIR absorption spectrum of the oligo- and poly(PADPA) reaction products obtained at pH = 4.3 after t = 24 h at RT with HRPC/H_2O_2 either in the presence of SDBS micelles in HEPES solution (1, black line, this work), or in the presence of AOT large unilamellar vesicles in dihydrogenphosphate solution (2, red line, previous work).[S6]

Reaction conditions:
[HRPC] = 30 nM; $[PADPA]_0$ = 1.0 mM; $[H_2O_2]_0$ = 1.0 mM
1: 0.1 M HEPES, pH = 4.3; [SDBS] = 3.0 mM (this work), see Fig. 3.
2: 0.1 M $H_2PO_4^-$, pH = 4.3; [AOT] = 2.0 mM (previous work, see Luginbühl et al. (2017)[S6]

.

Comparison of the UV/vis/NIR absorption spectrum of reaction mixtures containing SDBS micelles, PADPA, H_2O_2, and either hemin or HRPC prepared with either HEPES or dihydrogenphosphate solution at pH = 4.3, reacted for t = 24h at RT

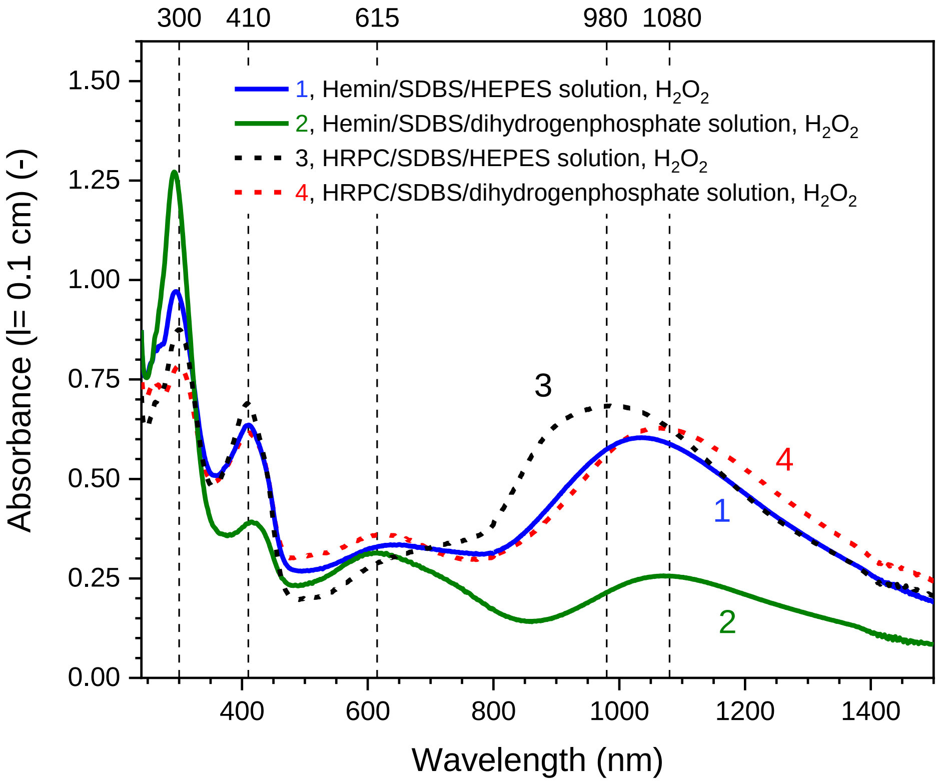

Figure S-5 Influence of the salt solution used for the hemin- or HRPC-catalyzed oxidation of PADPA at pH = 4.3 in the presence of H_2O_2, using either a HEPES or a dihydrogenphosphate solution. Comparison of the UV/vis/NIR absorption spectrum after t = 24 h at RT.

Reaction conditions:
$[PADPA]_0$ = 1.0 mM; $[H_2O_2]_0$ = 1.0 mM
1: 0.1 M HEPES, pH = 4.3; [hemin] = 10 μM, [SDBS] = 5.0 mM
2: 0.1 M $H_2PO_4^-$, pH = 4.3; [hemin] = 10 μM, [SDBS] = 5.0 mM
3: 0.1 M HEPES, pH = 4.3; [HRPC] = 30 nM, [SDBS] = 3.0 mM
4: 0.1 M $H_2PO_4^-$, pH = 4.3; [HRPC] = 30 nM, [SDBS] = 3.0 mM

Note:
In the case of hemin, the high concentration used (10 μM) contributes to the absorption at λ = 400 nm with A_{400} (0.1 cm) $\leq$ 0.066 (see Fig. S-3).

Comparison of the activity of hemin in the presence of SDBS micelles, H_2O_2 and either 0.1 M HEPES or 0.1 M dihydrogenphosphate at pH = 4.3 using the TMB assay

TMB assay[S7]

The assay was carried out in disposable polystyrene cuvettes with a pathlength l = 1 cm. The oxidation of TMB (3,3',5,5'-tetramethylbenzidine) to the TMB radical cation (one-electron oxidation) is followed by measuring the initial increase in A_{652} after mixing all reaction components. These components were added in the following sequence: (1) aqueous HEPES or dihydrogenphosphate solution, (2) SDBS stock solution, (3) hemin stock solution (in DMSO), (4) TMB stock solution, and (5) H_2O_2 stock solution After addition of each component, the reaction mixture was mixed. The final volume was always 1 mL. The UV/vis spectrum of the reaction mixture was recorded every 5 s during the course of 350 s at RT by using a diode array spectrophotometer (Specord S 600). For each condition, three measurements were carried out.

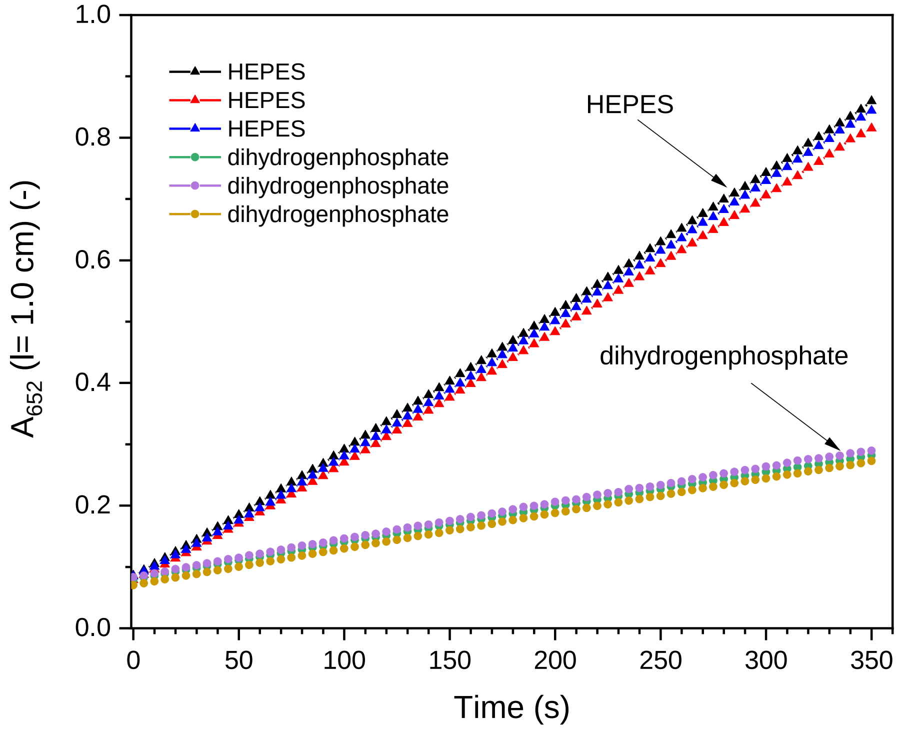

Figure S-6 TMB assay carried out for the hemin system either in the presence of 0.1 M HEPES or 0.1 M dihydrogenphosphate solution at pH = 4.3 and RT.

Assay conditions:

[SDBS]= 15.0 mM; [hemin] = 10 µM; $[TMB]_0$ = 0.3 mM; $[H_2O_2]_0$ = 0.3 mM. The UV/vis spectrum was recorded every 5 s during the course of 350 s after starting the reaction.

Note:

Despite the fact that the SDBS concentration used in the TMB assay was not the same as the one used for the reaction with PADPA, important was a direct comparison of the influence of the salt type used on the activity of hemin under otherwise identical conditions.

Control measurements for the HRPC/HEPES system

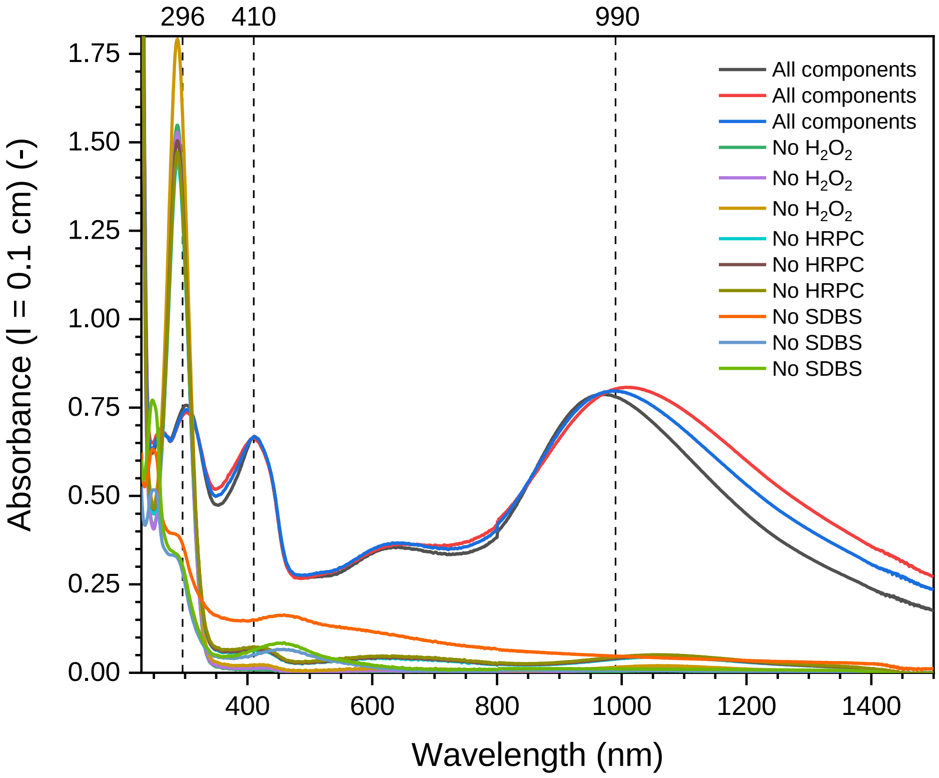

Figure S-7 Effect of the absence of one of the components of the "optimal reaction mixture" with HRPC as catalyst in 0.1 M HEPES pH = 4.3 solution, run in triplicates for t = 24 h at RT.

Reaction conditions:
All components: [HRPC] = 30 nM; $[PADPA]_0$ = 1.0 mM; [SDBS] = 3.0 mM; $[H_2O_2]_0$ = 1.0 mM
No H_2O_2: [HRPC] = 30 nM; $[PADPA]_0$ = 1.0 mM; [SDBS] = 3.0 mM
No HRPC: $[PADPA]_0$ = 1.0 mM; [SDBS] = 3.0 mM; $[H_2O_2]_0$ = 1.0 mM
No SDBS: [HRPC] = 30 nM; $[PADPA]_0$ = 1.0 mM; $[H_2O_2]_0$ = 1.0 mM

Note:
For the reaction without SDBS, product precipitation was observed, see also Luginbühl et al. (2017).[S6]

Control measurements for the hemin/HEPES system

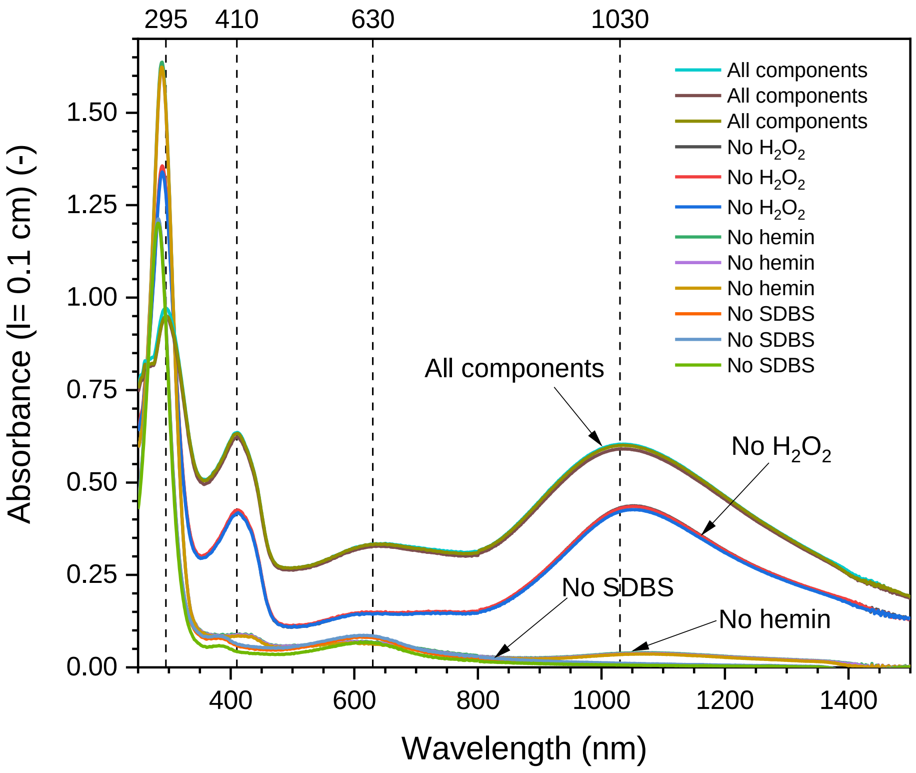

Figure S-8 Effect of the absence of one of the components of the "optimal reaction mixture" with hemin as catalyst in 0.1 M HEPES pH = 4.3 solution, run in triplicates for t = 24 h at RT.

Reaction conditions:
All components: [Hemin] = 10 μM; $[PADPA]_0$ = 1.0 mM; [SDBS] = 5.0 mM; $[H_2O_2]_0$ = 1.0 mM
No H_2O_2: [Hemin] = 10 μM; $[PADPA]_0$ = 1.0 mM; [SDBS] = 5.0 mM
No hemin: $[PADPA]_0$ = 1.0 mM; [SDBS] = 5.0 mM; $[H_2O_2]_0$ = 1.0 mM
No SDBS: [Hemin] = 10 μM; $[PADPA]_0$ = 1.0 mM; $[H_2O_2]_0$ = 1.0 mM

Notes:
(a) The high hemin concentration used (10 μM) contributes to the absorption at λ = 400 nm with A_{400} (0.1 cm) ≤ 0.066 (see Fig. S-3).
(b) For the reaction without SDBS, product precipitation was observed.

Comparison of the hemin/HEPES and HRPC/HEPES systems with and without H_2O_2

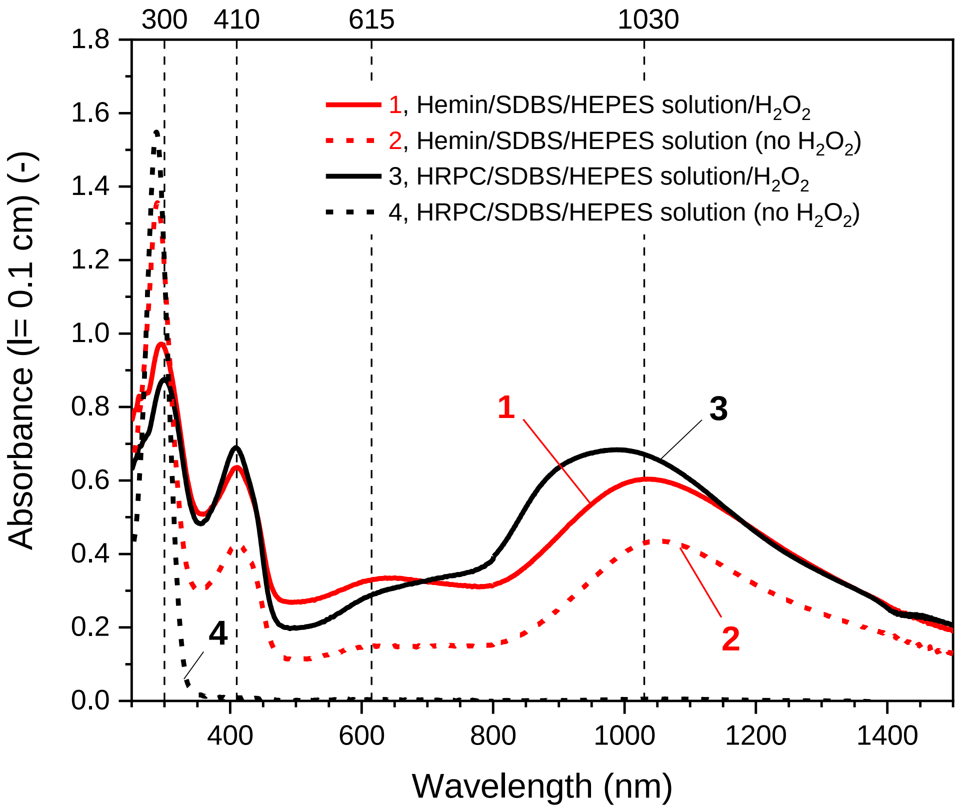

Figure S-9. Comparison of the UV/vis/NIR absorption spectra of the reaction mixtures containing either hemin (red) or HRPC (black) as catalyst, recorded after a reaction time of t = 24 h at RT, with (solid lines) and without (dashed lines) added H_2O_2.

Reaction conditions:
0.1 M HEPES, pH = 4.3; $[PADPA]_0$ = 1.0 mM
1: [Hemin] = 10 µM; [SDBS] = 5.0 mM; $[H_2O_2]_0$ = 1.0 mM
2: [Hemin] = 10 µM; [SDBS] = 5.0 mM
3: [HRPC] = 30 nM; [SDBS] = 3.0 mM; $[H_2O_2]_0$ = 1.0 mM
4: [HRPC] = 30 nM; [SDBS] = 3.0 mM

Note:
In the case of hemin, the high concentration used (10 µM) contributes to the absorption at λ = 400 nm with A_{400} (0.1 cm) $\leq$ 0.066 (see Fig. S-3).

Control measurements for the hemin/dihydrogenphosphate system

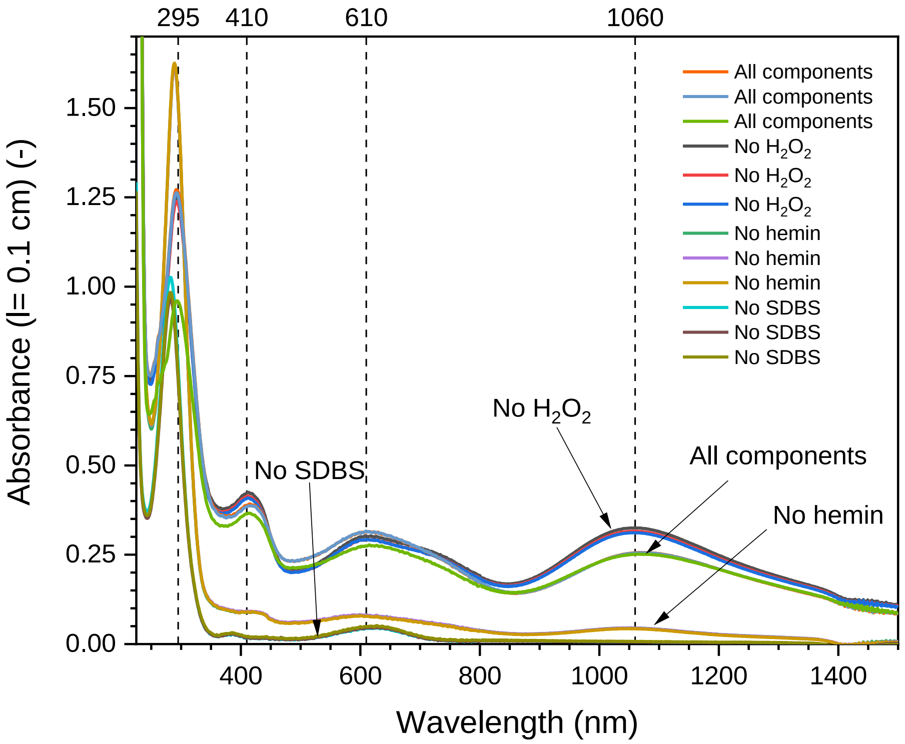

Figure S-10 Effect of the absence of one of the components of the reaction mixture with hemin as catalyst in 0.1 M $H_2PO_4^-$ pH = 4.3 solution, run in triplicates for t = 24 h at RT.

Reaction conditions:

All components: [Hemin] = 10 μM; $[PADPA]_0$ = 1.0 mM; [SDBS] = 5.0 mM; $[H_2O_2]_0$ = 1.0 mM
No H_2O_2: [Hemin] = 10 μM; $[PADPA]_0$ = 1.0 mM; [SDBS] = 5.0 mM
No hemin: $[PADPA]_0$ = 1.0 mM; [SDBS] = 5.0 mM; $[H_2O_2]_0$ = 1.0 mM
No SDBS: [Hemin] = 10 μM; $[PADPA]_0$ = 1.0 mM; $[H_2O_2]_0$ = 1.0 mM

Note:

(a) The high hemin concentration used (10 μM) contributes to the absorption at λ = 400 nm with A_{400} (0.1 cm) $\leq$ 0.066 (see Fig. S-3).
(b) For the reaction without SDBS, product precipitation was observed.

Control measurements for the HRPC/dihydrogenphosphate system

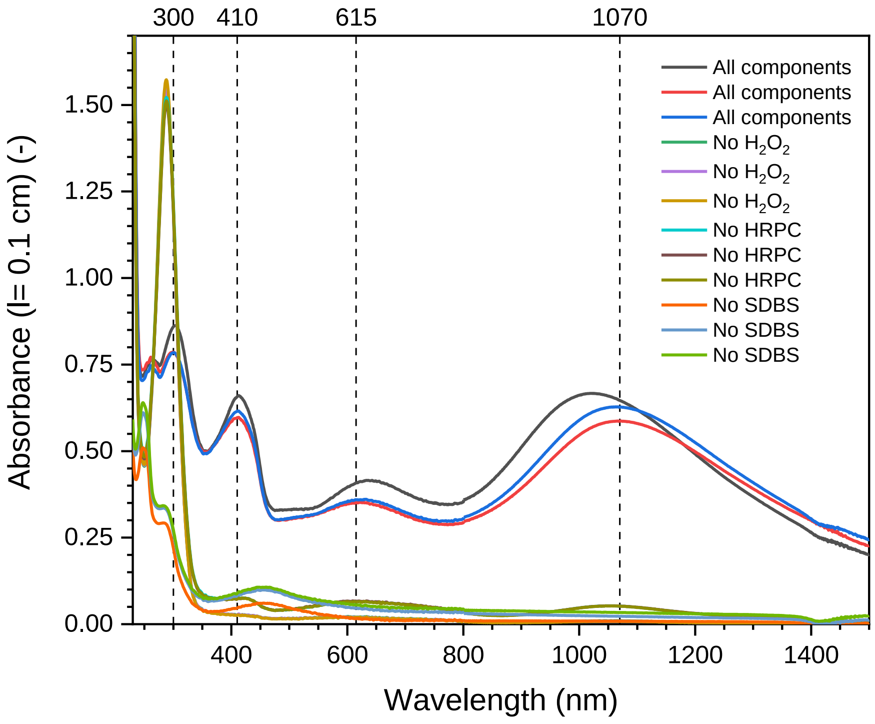

Figure S-11 Effect of the absence of one of the components of the reaction mixture with HRPC as catalyst in 0.1 M $H_2PO_4^-$ pH = 4.3 solution, run in triplicates for t = 24 h at RT.

Reaction conditions:
All components: [HRPC] = 30 nM; $[PADPA]_0$ = 1.0 mM; [SDBS] = 3.0 mM; $[H_2O_2]_0$ = 1.0 mM
No H_2O_2: [HRPC] = 30 nM; $[PADPA]_0$ = 1.0 mM; [SDBS] = 3.0 mM
No HRPC: $[PADPA]_0$ = 1.0 mM; [SDBS] = 3.0 mM; $[H_2O_2]_0$ = 1.0 mM
No SDBS: [HRPC] = 30 nM; $[PADPA]_0$ = 1.0 mM; $[H_2O_2]_0$ = 1.0 mM

Note:
For the reaction without SDBS, product precipitation was observed, see also Luginbühl et al. (2017).[S6]

Control measurements for the hemin/HEPES system using the TMB assay

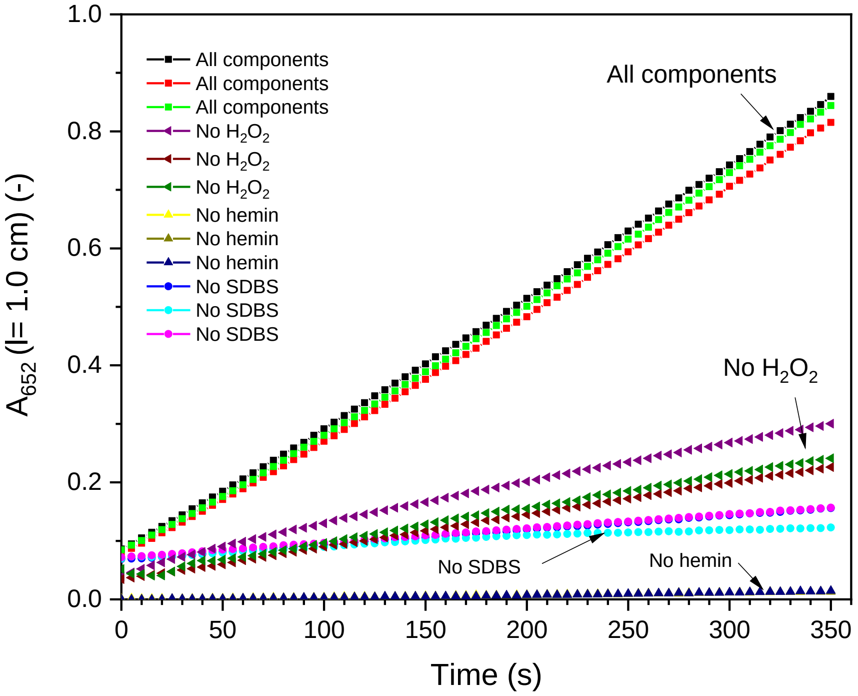

Figure S-12 TMB assay (see p. S-9) carried for the hemin/HEPES system in triplicates at RT, whereby the effect of omitting one of the components of the system was investigated. The UV/vis spectrum of the assay solution was recorded every 5 s during the course of 350 s after starting the reaction.

Assay conditions:
0.1 M HEPES solution, pH = 4.3; $[TMB]_0$ = 0.3 mM

All components: [Hemin] = 10 µM; [SDBS] = 15.0 mM; $[H_2O_2]_0$ = 0.3 mM
No H_2O_2: [Hemin] = 10 µM; [SDBS] = 15.0 mM
No hemin: [SDBS] = 15.0 mM; $[H_2O_2]_0$ = 0.3 mM
No SDBS: [Hemin] = 10 µM; $[H_2O_2]_0$ = 0.3 mM

Note:
Despite the fact that the SDBS concentration used in the TMB assay was not the same as the one used for the reaction with PADPA, important was a direct comparison of the influence of the salt type used on the activity of hemin under otherwise identical conditions.

Control measurements for the hemin/dihydrogenphosphate system using the TMB assay

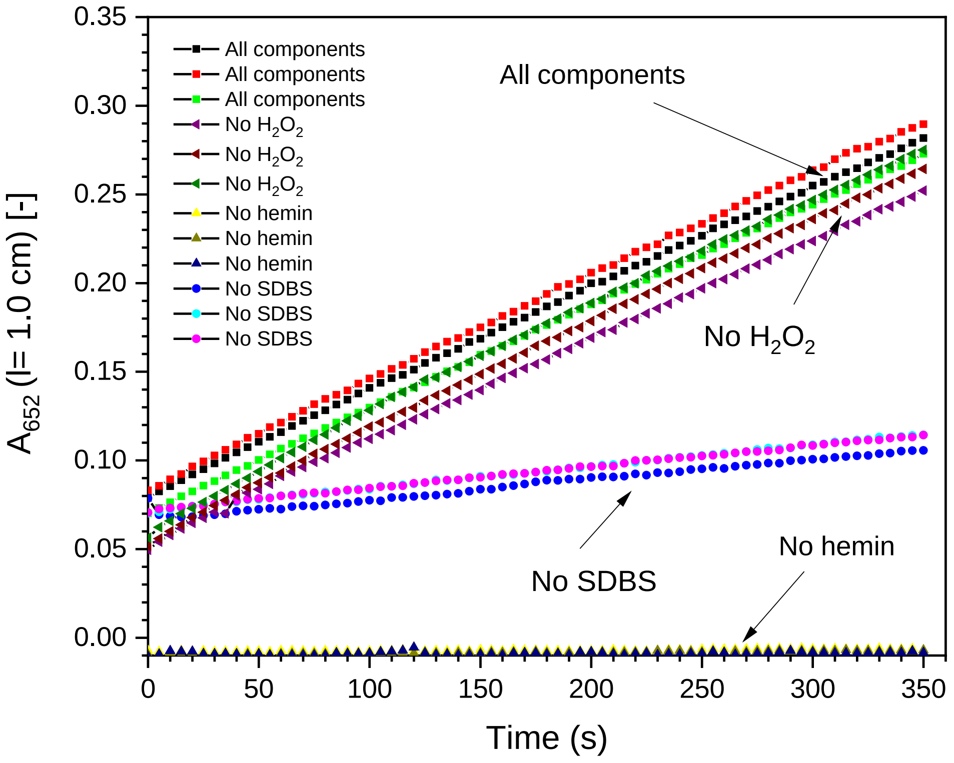

Figure S-13 TMB assay (see p. S-9) carried for the hemin/dihydrogenphosphate system in triplicates at RT, whereby the effect of omitting one of the components of the system was investigated. The UV/vis spectrum of the assay solution was recorded every 5 s during the course of 350 s after starting the reaction.

Assay conditions:
0.1 M $H_2PO_4^-$ solution, pH = 4.3; $[TMB]_0$ = 0.3 mM

All components: [Hemin] = 10 μM; [SDBS] = 15.0 mM; $[H_2O_2]_0$ = 0.3 mM
No H_2O_2: [Hemin] = 10 μM; [SDBS] = 15.0 mM
No hemin: [SDBS] = 15.0 mM; $[H_2O_2]_0$ = 0.3 mM
No SDBS: [Hemin] = 10 μM; $[H_2O_2]_0$ = 0.3 mM

Note:
Despite the fact that the SDBS concentration used in the TMB assay was not the same as the one used for the reaction with PADPA, important was a direct comparison of the influence of the salt type used on the activity of hemin under otherwise identical conditions.

Time-dependent changes of the UV/vis/NIR absorption spectrum of the reaction mixture prepared with the <u>HEPES</u> solution at pH = 4.3 and at RT, containing <u>hemin</u>, PADPA, <u>SDBS</u> micelles and <u>H_2O_2</u> ("optimal reaction conditions" for hemin)

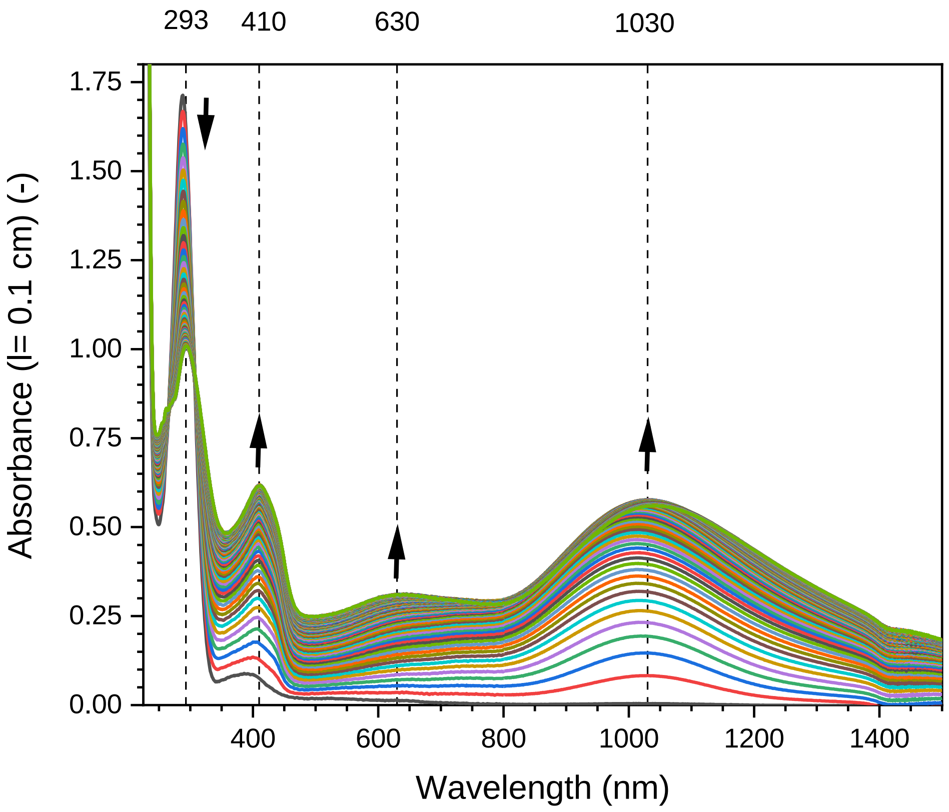

Figure S-14 Time-dependent changes of the UV/vis/NIR absorption spectrum for the formation of oligo- and poly(PADPA) products from PADPA in HEPES solution with hemin as catalyst under "optimal reaction conditions".

Reaction conditions:
0.1 M HEPES solution, pH = 4.3; [hemin] = 10 μM; [SDBS] = 5.0 mM; $[PADPA]_0$ = 1.0 mM; $[H_2O_2]_0$ = 1.0 mM. The spectrum was recorded every 15 min up to t ≈ 24 h; RT.

Time-dependent changes of the UV/vis/NIR absorption spectrum of the reaction mixture prepared with the HEPES solution at pH = 4.3 and at RT, containing HRPC, PADPA, SDBS micelles and H_2O_2 ("optimal reaction conditions" for HRPC)

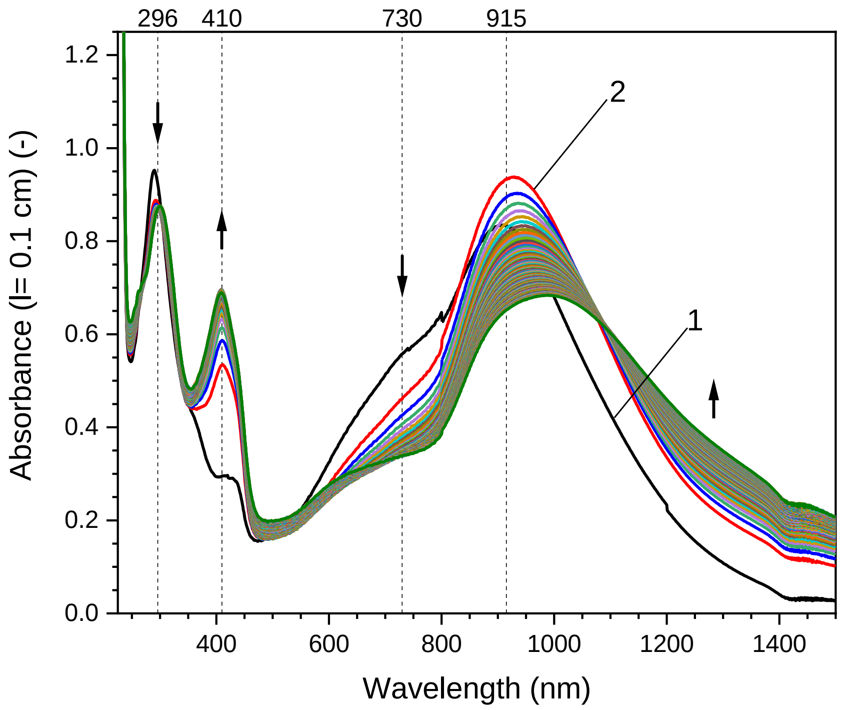

Figure S-15 Time-dependent changes of the UV/vis/NIR absorption spectrum for the formation of oligo- and poly(PADPA) products from PADPA in HEPES solution with HRPC as catalyst under "optimal reaction conditions".

Reaction conditions:
0.1 M HEPES solution, pH = 4.3; [HRPC] = 30 nM; [SDBS] = 3.0 mM; $[PADPA]_0$ = 1.0 mM; $[H_2O_2]_0$ = 1.0 mM. The spectrum was recorded every 15 min up to t ≈ 24 h (1: initial spectrum; 2: spectrum recorded after t = 30 min); RT.

Time-dependent changes of the UV/vis/NIR absorption spectrum of the reaction mixture prepared with the <u>HEPES</u> solution at pH = 4.3 and at RT, containing <u>hemin</u>, PADPA, and <u>SDBS</u> micelles <u>without H_2O_2</u>.

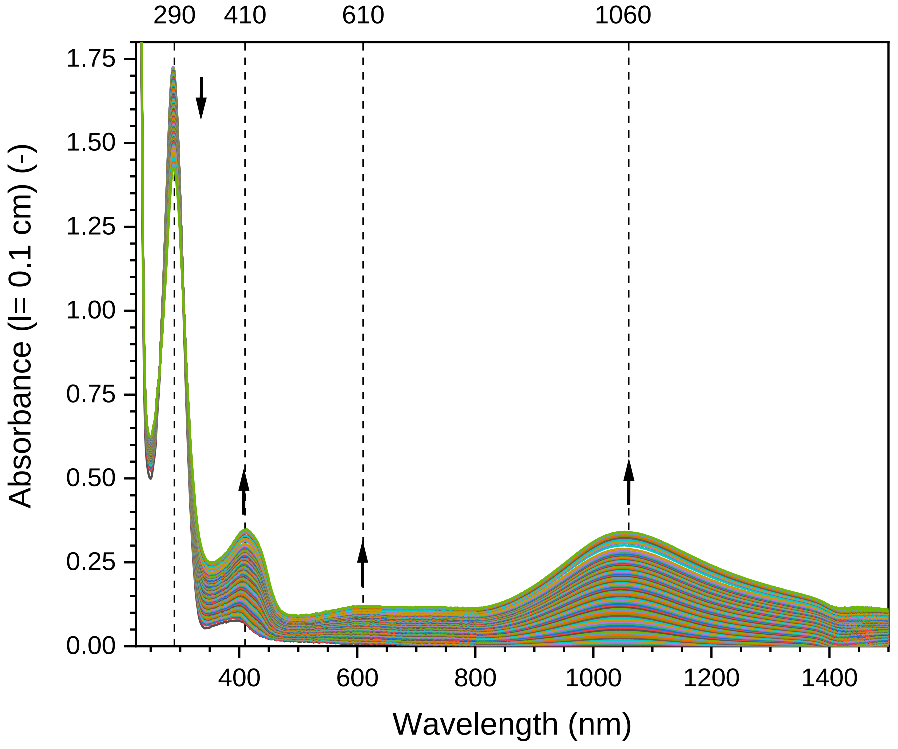

Figure S-16 Time-dependent changes of the UV/vis/NIR absorption spectrum upon reaction of PADPA in HEPES solution with hemin as catalyst *without* H_2O_2.

Reaction conditions:
0.1 M HEPES solution, pH = 4.3; [hemin] = 10 μM; [SDBS] = 5.0 mM; $[PADPA]_0$ = 1.0 mM. The spectrum was recorded every 15 min up to t ≈ 24 h; RT.

Time-dependent changes of the UV/vis/NIR absorption spectrum of the reaction mixture prepared with the dihydrogenphosphate solution at pH = 4.3 and at RT, containing hemin, PADPA, SDBS micelles and H_2O_2

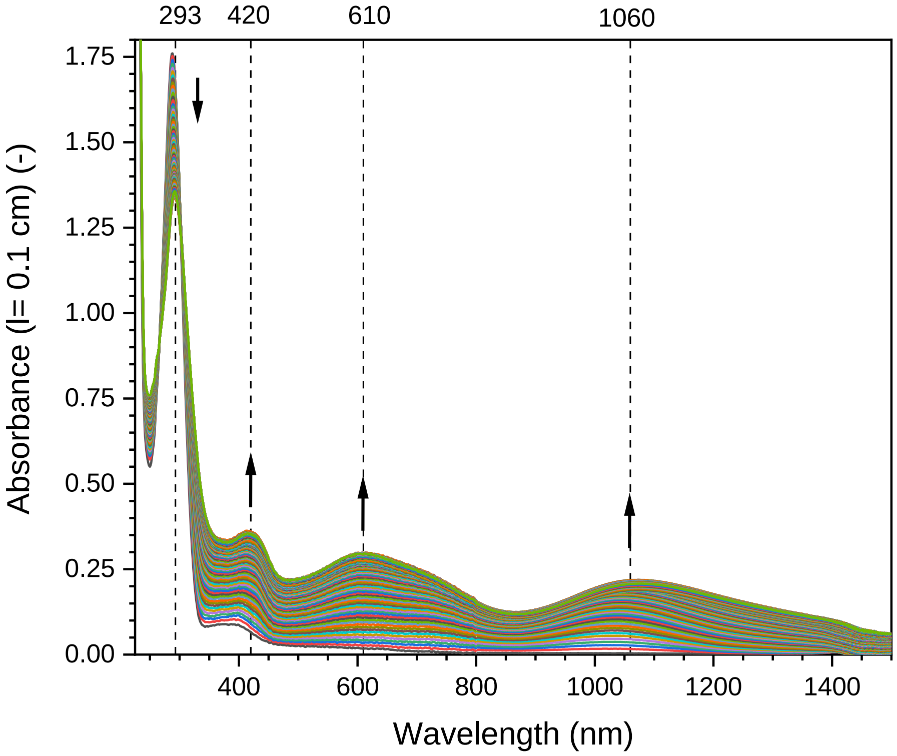

Figure S-17 Time-dependent changes of the UV/vis/NIR absorption spectrum upon reaction of PADPA in *dihydrogenphosphate* solution with hemin as catalyst.

Reaction conditions:
0.1 M $H_2PO_4^-$ solution, pH = 4.3; [hemin] = 10 μM; [SDBS] = 5.0 mM; $[PADPA]_0$ = 1.0 mM; $[H_2O_2]_0$ = 1.0 mM. The spectrum was recorded every 15 min up to t ≈ 24 h; RT.

Time-dependent changes of the UV/vis/NIR absorption spectrum of the reaction mixture prepared with the dihydrogenphosphate solution at pH = 4.3 and at RT, containing hemin, PADPA, and SDBS micelles without H_2O_2.

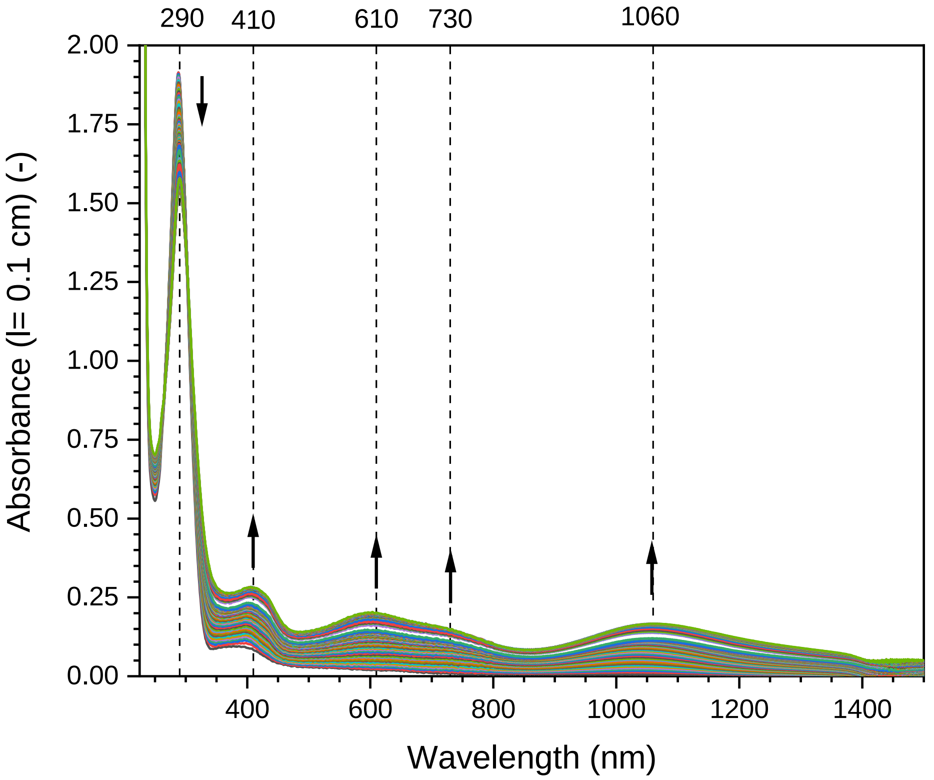

Figure S-18 Time-dependent changes of the UV/vis/NIR absorption spectrum upon reaction of PADPA in *dihydrogenphosphate* solution with hemin as catalyst *without* H_2O_2.

Reaction conditions:
0.1 M $H_2PO_4^-$ solution, pH = 4.3; [hemin] = 10 μM; [SDBS] = 5.0 mM; $[PADPA]_0$ = 1.0 mM. The spectrum was recorded every 15 min up to t ≈ 24 h; RT.

Comparison of the time-dependent changes of A_{1030} upon oxidation of PADPA in different reaction mixtures containing hemin or HRPC as catalyst at pH = 4.3 and RT

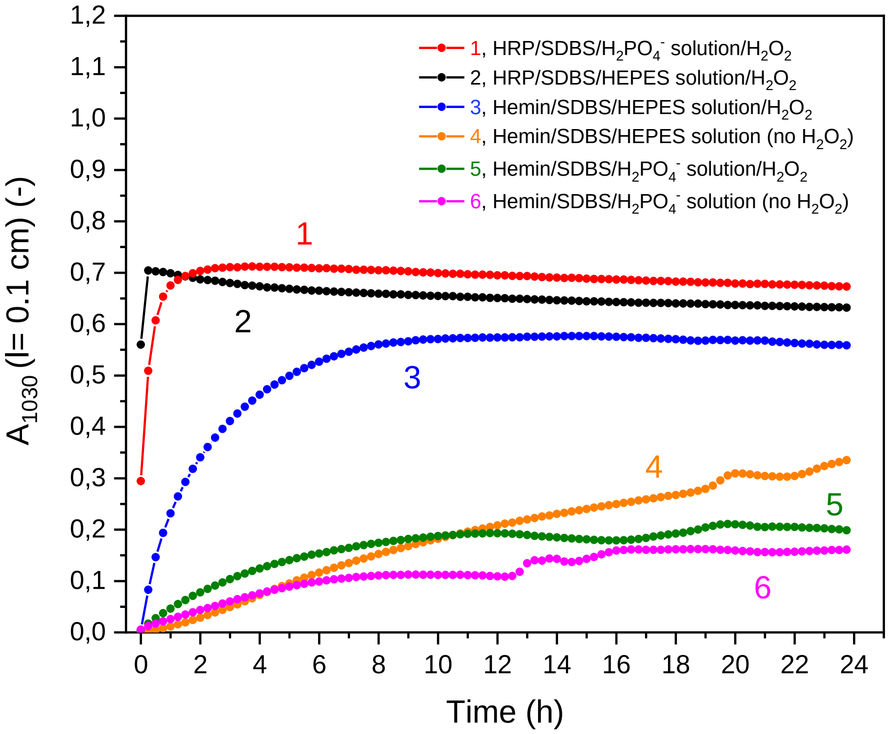

Figure S-19 Comparison of the changes of A_{1030} during the reaction of PADPA in different reaction mixtures. The entire spectrum was recorded every 15 min up to t ≈ 24 h and A_{1030} was plotted as a function of reaction time; RT.

Reaction conditions:
$[PADPA]_0$ = 1.0 mM, pH = 4.3
1: 0.1 M $H_2PO_4^-$; [HRPC] = 30 nM; [SDBS] = 3.0 mM; $[H_2O_2]_0$ = 1.0 mM
2: 0.1 M HEPES; [HRPC] = 30 nM; [SDBS] = 3.0 mM; $[H_2O_2]_0$ = 1.0 mM
3: 0.1 M HEPES; [Hemin] = 10 μM; [SDBS] = 5.0 mM; $[H_2O_2]_0$ = 1.0 mM ("optimal conditions")
4: 0.1 M HEPES; [Hemin] = 10 μM; [SDBS] = 5.0 mM
5: 0.1 M $H_2PO_4^-$; [Hemin] = 10 μM; [SDBS] = 5.0 mM; $[H_2O_2]_0$ = 1.0 mM
6: 0.1 M $H_2PO_4^-$; [Hemin] = 10 μM; [SDBS] = 5.0 mM

Note:
The data for 2, 3, and 4 are the same as the ones shown in Fig. 5.

3. EPR spectroscopy measurements

Analysis of reaction mixtures prepared with 0.1 M dihydrogenphosphate, pH = 4.3

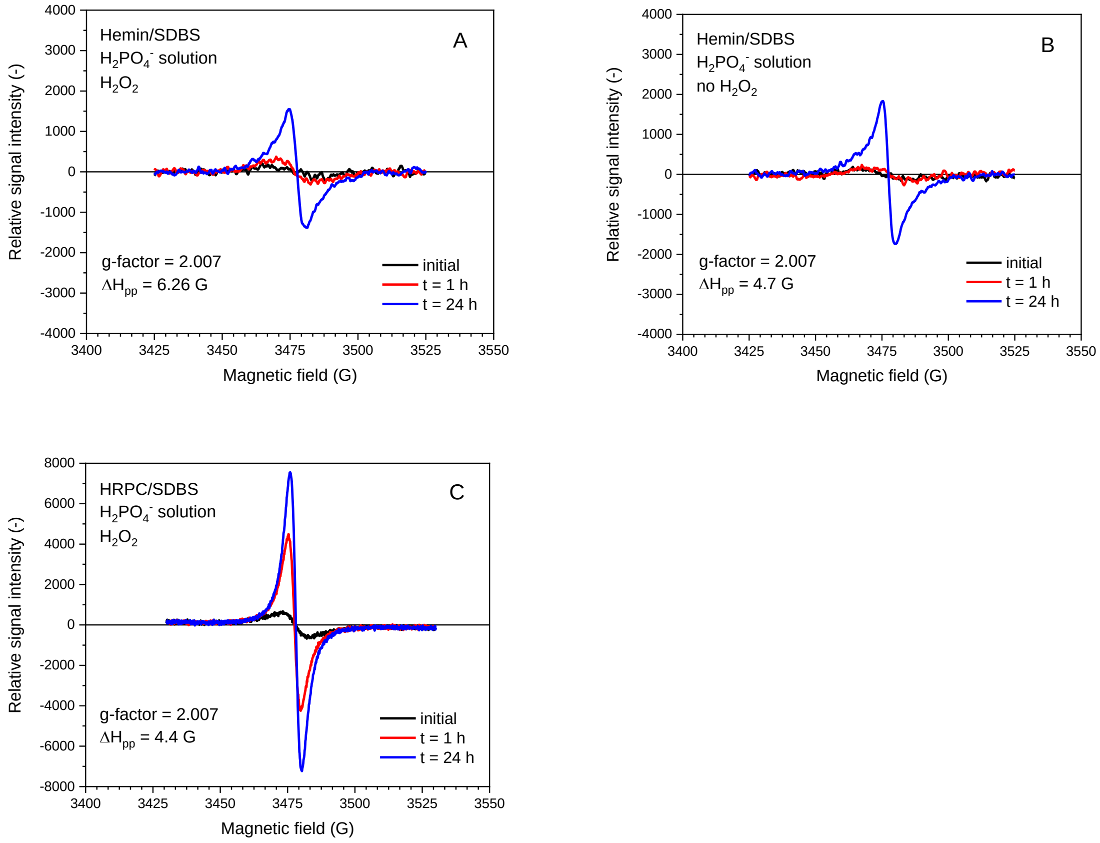

Figure S-20 EPR spectroscopy measurements of reaction mixtures containing SDBS micelles, prepared at RT with 0.1 M *dihydrogenphosphate*, pH = 4.3, with either hemin (A and B) or HRPC (C) as catalyst; either with (A and C) or without (B) H_2O_2.

Reaction conditions:
0.1 M $H_2PO_4^-$, pH = 4.3; $[PADPA]_0$ = 1.0 mM,
A: [Hemin] = 10 µM; [SDBS] = 5.0 mM; $[H_2O_2]_0$ = 1.0 mM
B: [Hemin] = 10 µM; [SDBS] = 5.0 mM
C: [HRPC] = 30 nM; [SDBS] = 3.0 mM; $[H_2O_2]_0$ = 1.0 mM

Observations:
The calculated g-factors are identical to the ones obtained in the counterpart systems containing 0.1 M HEPES (Fig. 6 and Fig. 7) instead of 0.1 M dihydrogenphosphate salt. The EPR signal width (ΔH_{pp}) is similar when comparing the HRPC system (C) with the hemin system *without* added H_2O_2 (B). Less uniform radical species are observed in the hemin-catalyzed system *with* added H_2O_2.

4. Raman spectroscopy measurements

Raman spectroscopy analysis of the oligo- and poly(PADPA) products obtained with hemin in the presence of SDBS micelles and H_2O_2, prepared with 0.1 M dihydrogenphosphate, pH = 4.3

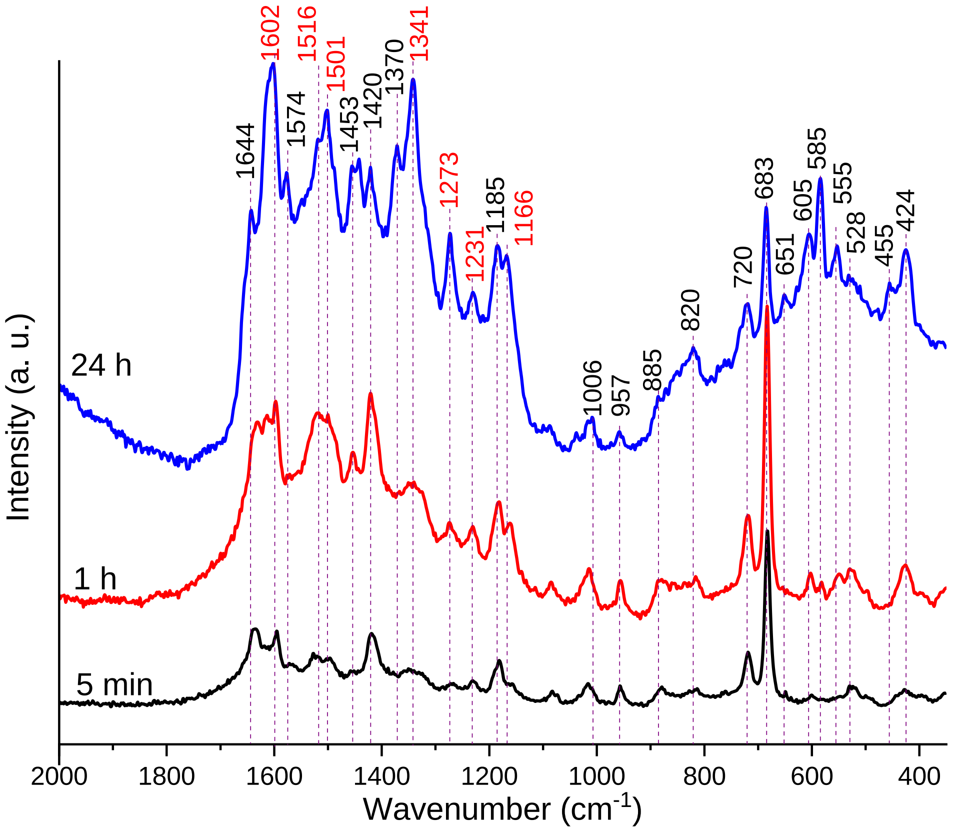

Figure S-21 Raman spectra of a reaction mixture containing hemin, SDBS micelles, PADPA, and H_2O_2, prepared with a 0.1 M dihydrogenphosphate solution, pH = 4.3, at RT. The spectra were recorded *in situ* after t = 5 min, t = 1 h, and t = 24 h. The bands characteristic for PANI-ES products are labeled in red; a.u., arbitrary units.

Reaction conditions:

0.1 M $H_2PO_4^-$, pH = 4.3; [Hemin] = 10 μM; [SDBS] = 5.0 mM; $[PADPA]_0$ = 1.0 mM, $[H_2O_2]_0$ = 1.0 mM

Raman spectroscopy analysis of the oligo- and poly(PADPA) products obtained with <u>hemin</u> in the presence of <u>SDBS</u> micelles <u>without H_2O_2</u>, prepared with 0.1 M <u>dihydrogenphosphate</u>, pH = 4.3

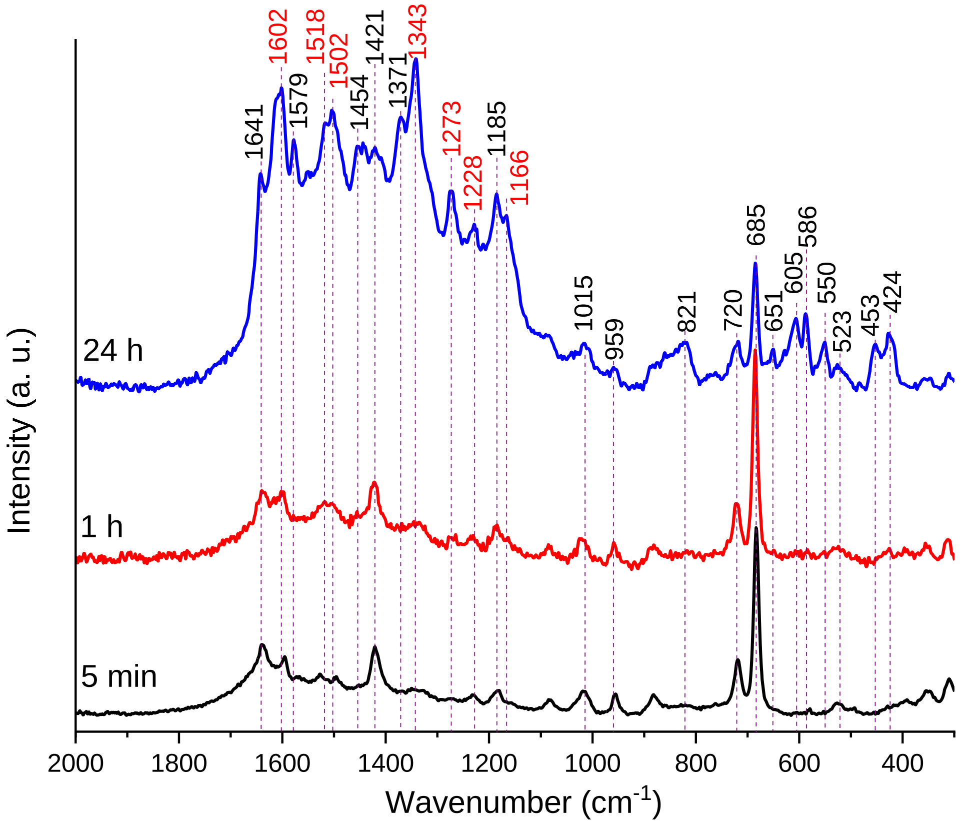

Figure S-22 Raman spectra of a reaction mixture containing hemin, SDBS micelles, PADPA, *without* H_2O_2, prepared with a 0.1 M dihydrogenphosphate solution, pH = 4.3, at RT. The spectra were recorded *in situ* after t = 5 min, t = 1 h, and t = 24 h. The bands characteristic for PANI-ES products are labeled in red; a.u., arbitrary units.

Reaction conditions:

0.1 M $H_2PO_4^-$, pH = 4.3; [Hemin] = 10 μM; [SDBS] = 5.0 mM; [PADPA]$_0$ = 1.0 mM

Comparison of the Raman spectra recorded after a reaction time t = 24 h at RT of reaction mixtures containing hemin as catalyst, prepared either in HEPES or in dihydrogenphosphate solution, with or without H_2O_2

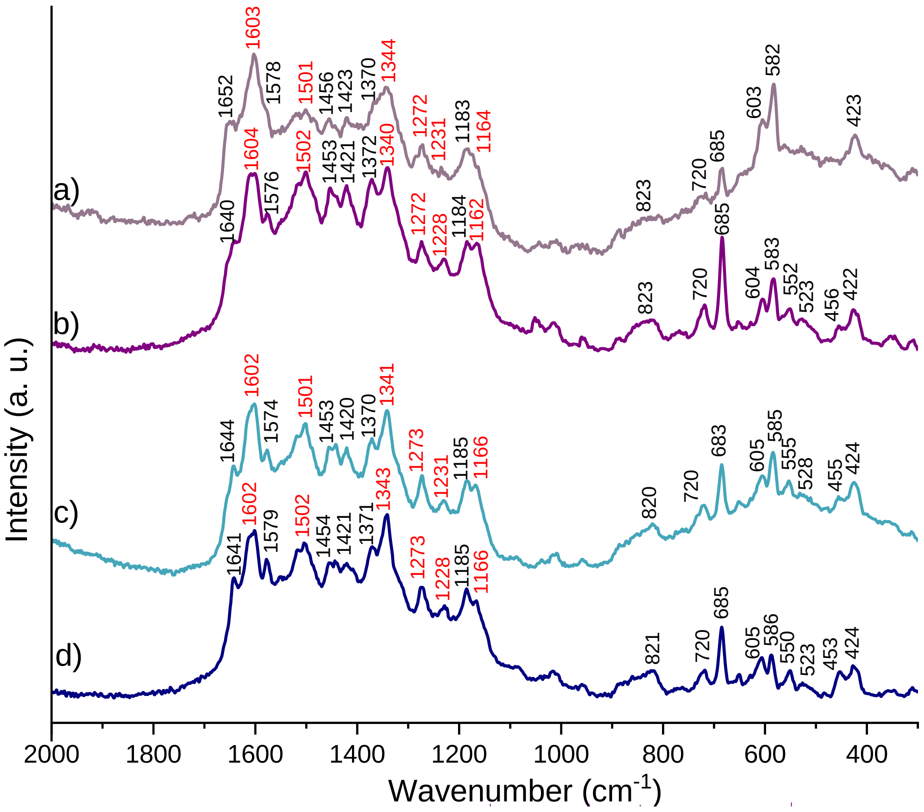

Figure S-23 Effect of salt type used and presence of H_2O_2 on the *in situ* recorded Raman spectrum of the oligo- and poly(PADPA) products obtained after t = 24 h at RT of reaction mixtures containing hemin with (a and c) or without (b and d) H_2O_2, prepared in HEPES solution (a and b) or in dihydrogenphosphate solution (c and d). The bands characteristic for PANI-ES products are labeled in red; a.u., arbitrary units.

Reaction conditions:

[SDBS] = 5.0 mM; [Hemin] = 10 μM; $[PADPA]_0$ = 1.0 mM

a) 0.1 M HEPES, pH = 4.3; $[H_2O_2]_0$ = 1.0 mM

b) 0.1 M HEPES, pH = 4.3

c) 0.1 M $H_2PO_4^-$, pH = 4.3; $[H_2O_2]_0$ = 1.0 mM

d) 0.1 M $H_2PO_4^-$, pH = 4.3

Raman spectroscopy analysis of the oligo- and poly(PADPA) products obtained with <u>HRPC</u> in the presence of <u>SDBS</u> micelles and <u>H_2O_2</u>, prepared with 0.1 M <u>dihydrogenphosphate</u>, pH = 4.3

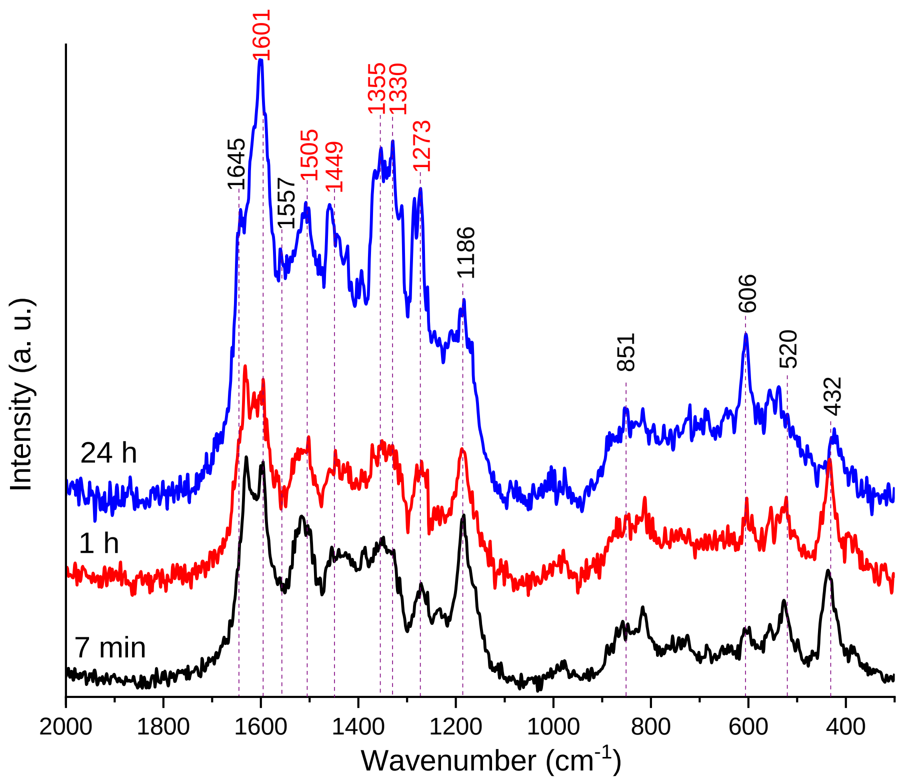

Figure S-24 Raman spectra of a reaction mixture containing HRPC, SDBS micelles, PADPA, and H_2O_2, prepared with a 0.1 M dihydrogenphosphate solution, pH = 4.3, at RT. The spectra were recorded *in situ* after t = 7 min, t = 1 h, and t = 24 h. The bands characteristic for PANI-ES products are labeled in red; a.u., arbitrary units.

Reaction conditions:

0.1 M $H_2PO_4^-$, pH = 4.3; [HRPC] = 30 nM; [SDBS] = 3.0 mM; $[PADPA]_0$ = 1.0 mM, $[H_2O_2]_0$ = 1.0 mM

5. Preliminary investigations of an "O_2-free" reaction mixture

From a reaction mixture containing hemin, SDBS micelles, and PADPA in HEPES solution (*without* H_2O_2), oxygen was removed and then the UV/vis/NIR absorption spectrum was recorded after 24 h. The conditions were as follows: aqueous 0.1 M aqueous HEPES solution at pH = 4.3, [SDBS] = 5.0 mM, [hemin] = 10 µM, and [PADPA] = 1.0 mM. The total reaction volume was 1 mL. All components were placed in a 50 mL round bottom flask (in the sequence mentioned) which was then connected to a Schlenk line. Oxygen was removed by three "freeze-pump-thaw" cycles. Such an "O_2-free" reaction mixture was covered with aluminum foil and left for 24 h. Then, a 350 µL aliquot was removed from the reaction mixture and placed in a quartz cuvette (l = 0.1 cm) and the UV/vis/NIR absorption spectrum was measured with a JASCO-V670 spectrophotometer. The spectrum obtained is shown in Figure S-25.

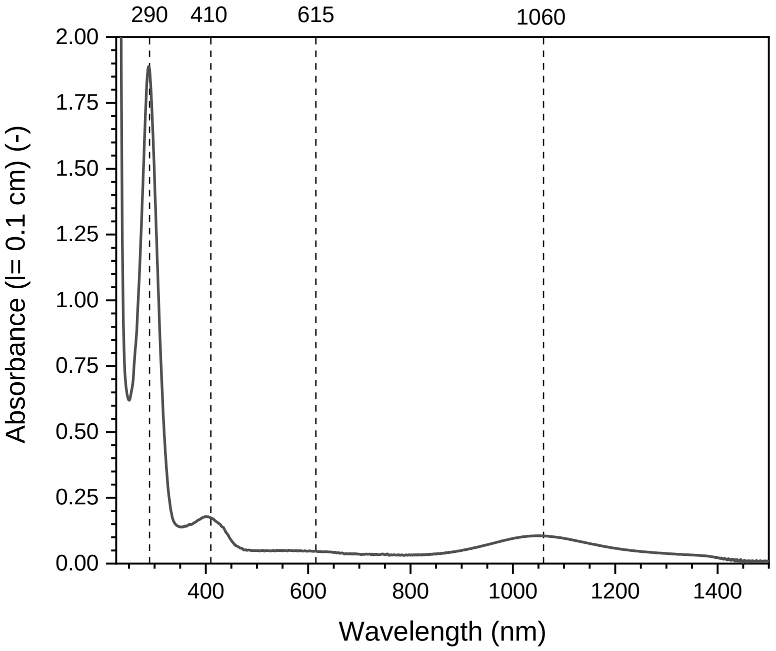

Fig. S-25 Reaction mixture composed of: 0.1 M aqueous HEPES solution at pH 4.3, [SDBS] = 5.0 mM, [hemin] = 10.0 µM and [PADPA] = 1.0 mM. After mixing all components, O_2 was removed, and the reaction mixture was kept in the dark under "O_2-free" conditions for 24 h, RT.

Note:
Since the components were added before oxygen was removed, the reaction could occur for a short period of time at the beginning. From this time on, the reaction proceeded to a much lower extent ($A_{1060} \approx 0.1$) compared to the same system (shown in Fig. S-8) where oxygen was present ($A_{1060} \approx 0.34$). A value of A_{1060} = 0.1 was reached in the reaction mixture *with* O_2 (but without H_2O_2) after t $\approx$ 5 h already (data extracted from Fig. S-16).

In order to further prove that the reaction was oxygen-dependent and that what we have observed is not due to some physicochemical changes of the components which might have occurred during the procedure of O_2-removal, the same reaction mixture of which the spectrum is shown in Fig. S-25 was first exposed to air and then mixed 10 times by turning the cuvette (closed with a stopper) up and down. After that, the cuvette was placed in the cuvette holder and the UV/vis/NIR absorption spectrum was measured every 15 minutes for the next 24 h using the JASCO-V670 instrument.

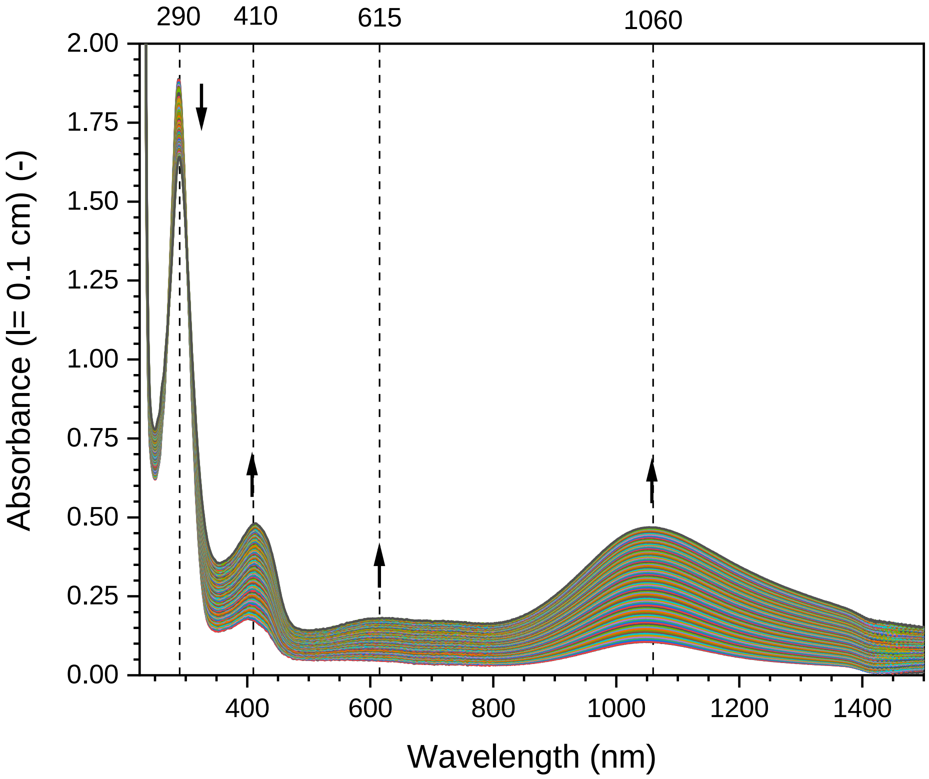

Figure S-26 UV-Vis-NIR spectra were recorded every 15 min during the course of 24 h, RT. The initial spectrum (the one with the lowest absorbance) is the spectrum that was recorded after the reaction has been carried out for 24 h in oxygen-free conditions (also shown in Fig. S-25). All of the spectra obtained after that one are the ones obtained from the same reaction mixture but in the presence of oxygen from air.

Observations:

As soon as the "O_2-free" reaction mixture got again in contact with oxygen from air, the reaction continued to proceed. This undoubtedly confirms that the observed reaction without added H_2O_2 is oxygen dependent and that the procedure of O_2-removal did not negatively affect the components of the reaction mixture.

6. Estimation of catalyst cost reduction when HRPC is replaced by hemin for the formation of PANI-ES products from PADPA

Catalyst amounts used
[**HRPC**] = 30 nM; [**Hemin**] = 10 μM
0.1 M HEPES solution, pH = 4.3; $[PADPA]_0$ = 1.0 mM; $[H_2O_2]_0$ = 1.0 mM
[SDBS] = 5.0 mM for the reaction with **hemin**
[SDBS] = 3.0 mM for the reaction with **HRPC**

*Costs for 1 L reaction mixture containing **HRPC***
Purchased from Toyobo Enzymes (through Sorachim SA, Switzerland), 20 kU, PEO-131
Date of purchase: March, 2018; LOT 8153665000; Price: 350 CHF (without delivery costs).
According to the certificate of analysis: 271 U/mg, Mw (HRPC) = 44 000 g/mol, i.e., 20 kU correspond to 74 mg HRPC.

4 mg PEO-131 dissolved in 1 mL of 0.1 M sodium phosphate buffer solution (pH = 7.0).
By measuring the absorbance at λ = 403 nm, A_{403}, and using ε_{403} = 102 000 $M^{-1}cm^{-1}$ (see Materials and methods) [HRPC] = 77.6 μM is obtained.
[HRPC] = 77.6 μM corresponds to 77.6 nmol HRPC per mL solution containing 4 mg HRPC.

[HRPC] = 30 nM in the reaction mixture (30 nmol/L) corresponds to 1.55 mg HRPC/L.
Price of 1.55 mg HRPC: 7.30 CHF

Costs of 30 nmol HRPC: 7.30 CHF

*Costs for 1 L reaction mixture containing **hemin***
Hemin from porcine, 1 gram, (≥97.0 % HPLC), purchased from Sigma.
Date of purchase: October, 2019
LOT BCCB6735
Price: 50 CHF
Mw (hemin) = 651.94 g/mol

Costs of 10 μmol hemin: 0.32 CHF

7. References

S1 K. Junker, I. Gitsov, N. Quade and P. Walde, *Chemical Papers*, 2013, **67**, 1028-1047.
S2 G. I. Berglund, G. H. Carlsson, A. T. Smith, H. Szöke, A. Henriksen and J. Hajdu, *Nature*, 2002, **417**, 463-468.
S3 E. Derat and S. Shaik, *Journal of the American Chemical Society*, 2006, **128**, 8185-8198.
S4 P. Campomanes, U. Rothlisberger, M. Alfonso-Prieto and C. Rovira, *Journal of the American Chemical Society*, 2015, **137**, 11170-11178.
S5 N. C. Veitch and A. T. Smith, in *Advances in Inorganic Chemistry*, Academic Press, 2000, vol. 51, pp. 107-162.
S6 S. Luginbühl, M. Milojević-Rakić, K. Junker, D. Bajuk-Bogdanović, I. Pašti, R. Kissner, G. Ćirić-Marjanović and P. Walde, *Synthetic Metals*, 2017, **226**, 89-103.
S7 P. D. Josephy, T. Eling and R. P. Mason, *Journal of Biological Chemistry*, 1982, **257**, 3669-3675.
